# Anterior expansion and posterior addition to the notochord mechanically coordinate zebrafish embryo axis elongation

**DOI:** 10.1242/dev.199459

**Published:** 2021-07-21

**Authors:** Susannah B. P. McLaren, Benjamin J. Steventon

**Affiliations:** Department of Genetics, University of Cambridge, Cambridge, CB2 3EH, UK

**Keywords:** Multi-tissue, Morphogenesis, Mechanics, Somitogenesis, Notochord, Vacuolation

## Abstract

How force generated by the morphogenesis of one tissue impacts the morphogenesis of other tissues to achieve an elongated embryo axis is not well understood. The notochord runs along the length of the somitic compartment and is flanked on either side by somites. Vacuolating notochord cells undergo a constrained expansion, increasing notochord internal pressure and driving its elongation and stiffening. Therefore, the notochord is appropriately positioned to play a role in mechanically elongating the somitic compartment. We used multi-photon cell ablation to remove specific regions of the zebrafish notochord and quantify the impact on axis elongation. We show that anterior expansion generates a force that displaces notochord cells posteriorly relative to adjacent axial tissues, contributing to the elongation of segmented tissue during post-tailbud stages. Unexpanded cells derived from progenitors at the posterior end of the notochord provide resistance to anterior notochord cell expansion, allowing for stress generation along the anterior-posterior axis. Therefore, notochord cell expansion beginning in the anterior, and addition of cells to the posterior notochord, act as temporally coordinated morphogenetic events that shape the zebrafish embryo anterior-posterior axis.

## Abstract (precis)

**Summary:** Targeted multi-photon tissue ablation reveals that coordinated cell expansion and addition to the notochord in zebrafish embryos contributes to the elongation of segmented tissue required for embryo anterior-posterior axis extension.

## INTRODUCTION

**Fig. 1.**
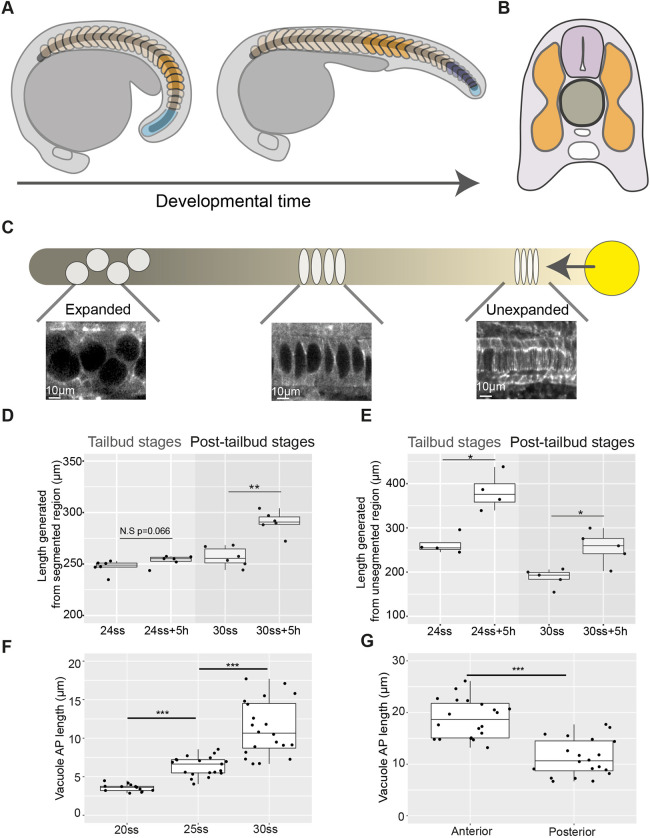
**Axis elongation occurs predominantly in the unsegmented region during tailbud stages and later continues in segmented tissue in zebrafish embryos.** (A) Schematic outlining segmented (orange and dark-blue) and unsegmented (light-blue) regions of the somitic compartment. Dark-blue somites are formed from unsegmented tissue as development progresses. (B) Schematic showing axial tissues in a cross-section of the zebrafish embryo. The notochord (dark-grey) is flanked on either side by the somitic compartment (orange) and dorsally by the neural tube (light-purple). (C) Schematic outlining events in notochord morphogenesis. Vacuolation progresses from the anterior (darker) to posterior (lighter) of the notochord and progenitors expressing *noto* (yellow) add cells to the notochord rod. (D) Length of a region of segmented tissue in tailbud (24ss) and post-tailbud (30ss) embryos and length generated from this region over 5 h (*n*=6 and *n*=6, respectively; **P<0.01, Mann–Whitney U test). (E) Length of a region of unsegmented tissue in tailbud (24ss) and post-tailbud (30ss) embryos and length generated from this region over 5 h (*n*=4 and *n*=5 embryos per stage, respectively; *P<0.05, Mann–Whitney U test). (F) AP length of vacuoles at different stages of development (*n*=3, *n*=4 and *n*=4 embryos per stage, respectively, 3-5 vacuoles measured per embryo; ***P<0.001, Kruskal–Wallis test). (G) AP length of vacuoles in anterior and posterior regions of 30ss embryos (*n*=4 embryos, 5 vacuoles measured per region; ***P<0.001, Mann–Whitney U test). Anterior is to the left and posterior to the right in all images. N.S., not significant.

Elongation of the embryo anterior-posterior (AP) axis requires the physical deformation of multiple axial tissues as they undergo morphogenesis (Bénazéraf et al., 2017; Steventon et al., 2016; Xiong et al., 2020). The vertebrate embryo body axis is segmented into blocks of tissue called somites, which later form the skeletal muscle and vertebrae of the adult body (Gomez et al., 2008). Running through the middle of the embryo, the rod-shaped notochord is flanked on either side by the somitic compartment: the somites in the segmented region of the axis and the presomitic mesoderm in the posterior (Fig. 1A,B) (Stemple, 2005).

Mutants lacking a notochord have fused somites (Talbot et al., 1995), and disruption of notochord morphogenesis results in axis truncation and skeletal malformations (Bagwell et al., 2020; Ellis et al., 2013). Studies in *Xenopus* embryos showed that the circumferentially constrained expansion of cells within the notochord sheath leads to an increase in notochord stiffness as the embryo develops, making it a candidate for driving the physical deformation of surrounding somitic tissue that may be required for AP axis elongation (Adams et al., 1990; Koehl et al., 2000). In the zebrafish notochord, cells differentiate into either a sheath or vacuolated cell type (Yamamoto et al., 2010). Vacuolating notochord cells expand over time as they draw in fluid, as in *Xenopus* (Fig. 1C) (Ellis et al., 2013), and notochord volume increases as development progresses (Steventon et al., 2016). However, it remains unclear how force generated by notochord morphogenesis impacts the elongation of the somitic compartment and extends the zebrafish embryo AP axis.

Concomitant with notochord cell expansion, a separate morphogenetic event occurs, in which cells are contributed to the posterior end of the notochord from a population of notochord progenitors (Fig. 1C) (Kanki and Ho, 1997). Early termination of progenitor addition to the notochord is associated with AP axis elongation defects (Row et al., 2016). Although the role of other tailbud-located progenitor populations in elongating the AP axis has been investigated (Goto et al., 2017; Mongera et al., 2018; Attardi et al., 2018), it is not known how the posterior contribution of cells from notochord progenitors is coordinated with the expansion of more-anterior notochord cells, and how these combined processes impact axis elongation.

As notochord morphogenesis progresses, somites are segmented from the unsegmented presomitic mesoderm in the posterior tailbud until 30-34 somites are formed in total, with the tailbud remaining present until the 30-somite stage (30ss), when it begins to degenerate (Fig. 1A) (Gomez et al., 2008; Oates et al., 2012; Kimmel et al., 1995). As somites mature, they undergo bending until a ‘chevron shape’ is achieved (Rost et al., 2014). Although elongation generated from unsegmented tailbud-derived tissue has been studied (Das et al., 2019; Dray et al., 2013; Lawton et al., 2013; Mongera et al., 2018; Steventon et al., 2016), how elongation progresses in mature segmented tissue during post-tailbud stages of development is less clear.

Here, we used targeted multi-photon tissue ablation to investigate the physical impact of notochord morphogenesis on the surrounding somitic compartment. We identify the concomitant expansion of notochord cells and contribution of progenitors to the posterior end of the notochord as two temporally coordinated morphogenetic events that shape the zebrafish embryo AP axis.

## RESULTS AND DISCUSSION

### Modes of axis elongation change as embryos progress from tailbud stages into post-tailbud stages of development

To characterise quantitatively AP axis elongation occurring concomitantly with notochord volume increase, we live-imaged developing zebrafish embryos as notochord cell expansion was progressing and quantified elongation in segmented and unsegmented regions of the axis (Fig. S1A,B; Movie 1). Segmented tissue elongation was tracked using the boundaries of five formed somites and segmentation-associated elongation was tracked using the presomitic mesoderm and somites formed as development progressed (Fig. 1A).

We compared elongation over a 5 h period during tailbud (24ss) and post-tailbud stages (30ss) to investigate the modes of axis elongation during each of these phases of embryo morphogenesis. No significant increase in segmented tissue length was measured during tailbud stages (Fig. 1D; Fig. S1A). However, in post-tailbud stages mean segmented tissue length increased by ∼13% (Fig. 1D; Fig. S1B). In contrast, less segmentation-associated elongation was measured in post-tailbud stages compared with tailbud-stage embryos (Fig. 1E; Fig. S1A,B).

These findings show that embryo AP axis elongation can be described by two phases: segmentation-associated elongation during tailbud stages, followed by elongation of segmented tissue during post-tailbud stages (Movie 1).

### Notochord cell expansion leads to the posterior displacement of notochord cells relative to adjacent axial tissues

**Fig. 2.**
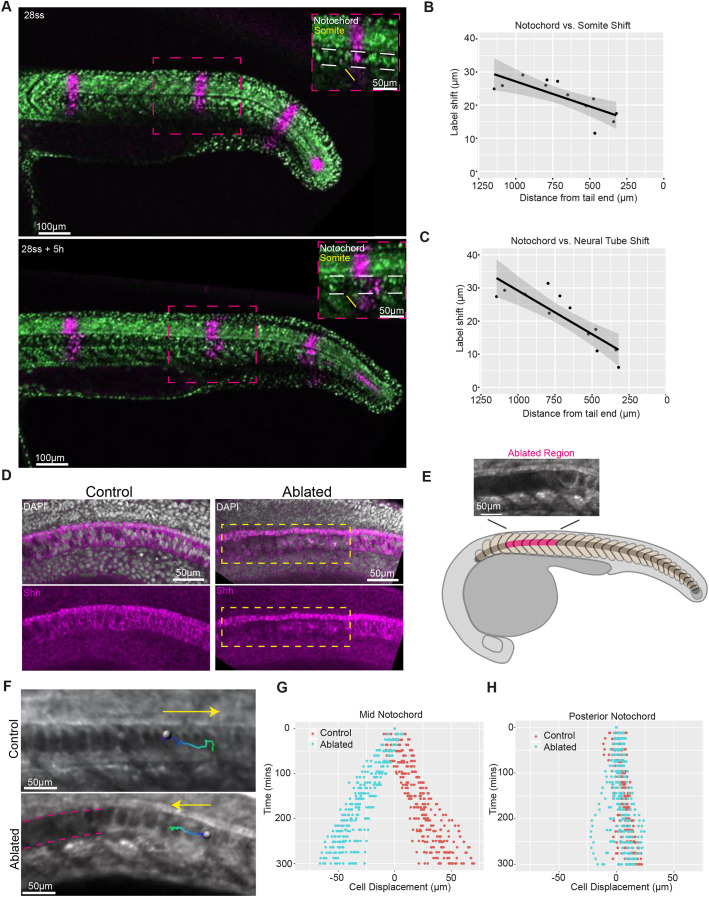
**Notochord cell expansion leads to the posterior displacement of notochord cells relative to adjacent axial tissues in zebrafish embryos.** (A) Photolabelled stripes (magenta) along the length of an embryo expressing NLS-KikGR at 28ss and 5 h later. White dashed lines in insets show the notochord. (B) Shift between notochord and somite photolabels versus distance from the posterior end of the embryo. (C) Shift between notochord and neural tube photolabels versus distance from the posterior end of the embryo. Black dots indicate measured label shifts, solid line indicates linear regression line and grey shading indicates 95% confidence interval. (D) DAPI and Shh expression in representative control and ablated embryos (*n*=4 embryos per condition). The boxed area indicates the ablated region. (E) Schematic showing the ablated region (pink) in an embryo entering the post-tailbud stage. Ablations were made at the onset of vacuolation. (F) Expanding notochord cell tracks (track start: dark blue; track end: light green) in control and ablated embryos. Yellow arrows indicate direction of tracks. Pink dashed lines indicate ablated region. (G) Displacement of manually tracked expanded notochord cells in control and ablated embryos (*n*=6 embryos per condition, 3 tracks per embryo). (H) Displacement of manually tracked unexpanded notochord cells in control and ablated embryos (*n*=5 embryos per condition, 3 tracks per embryo).

To gain insight into how notochord morphogenesis impacts axis elongation during tailbud and post-tailbud stages of development, we characterised notochord cell expansion both temporally, in an equivalent region of the notochord of different-stage embryos (Fig. S2A), and spatially, in notochord regions in the anterior versus posterior of embryos at the same developmental stage (Fig. S2B). In agreement with previous work (Bagwell et al., 2020; Dale and Topczewski, 2011; Yamamoto et al., 2010), we observed that vacuolating notochord cells expanded over time (Fig. 1F), with the earlier onset of vacuolation in the anterior notochord likely leading to larger cell lengths in the anterior versus posterior (Fig. 1G). Photolabelling stripes along the axis in embryos entering post-tailbud stages revealed that anterior vacuolated notochord cells move posteriorly relative to adjacent regions of somitic compartment and neural tube (Fig. 2A), with the shift between notochord cells and adjacent tissues being smaller in the posterior region of the axis, where notochord cells are less expanded (Fig. 2B,C; Fig. S3).

To investigate how forces generated from anterior notochord cell expansion might propagate along the AP axis and to neighbouring tissues, a technique is required that allows for precise spatiotemporal removal of notochord cells. We used multi-photon ablation to cut through notochord cells at defined regions of the AP axis (Movie 2), resulting in the destruction of tissue local to the ablation site and minimal damage to adjacent tissues, such as the Shh-expressing floor plate lying immediately dorsal to the notochord (Fig. 2D). Ablations were performed at the onset of vacuolation, removing cells before water uptake and subsequent expansion (Fig. S2C). Ablation of anterior notochord cells reversed the direction of displacement of expanding vacuolated cells posterior to the ablation site (Fig. 2E-G; Movie 3) and, over longer timescales, the ablated region eventually closed, possibly aided by changes in cell packing (Norman et al., 2018) (Movie 4). However, the movement of unexpanded notochord cells in the posterior undifferentiated region was less affected (Fig. 2H). Thus, the expansion of vacuolated notochord cells, beginning in the anterior and progressing posteriorly along the notochord, generates a force that displaces neighbouring expanded notochord cells posteriorly relative to adjacent axial tissues in post-tailbud-stage embryos.

### Notochord cell expansion is a driver of segmented tissue elongation during post-tailbud stages of development

**Fig. 3.**
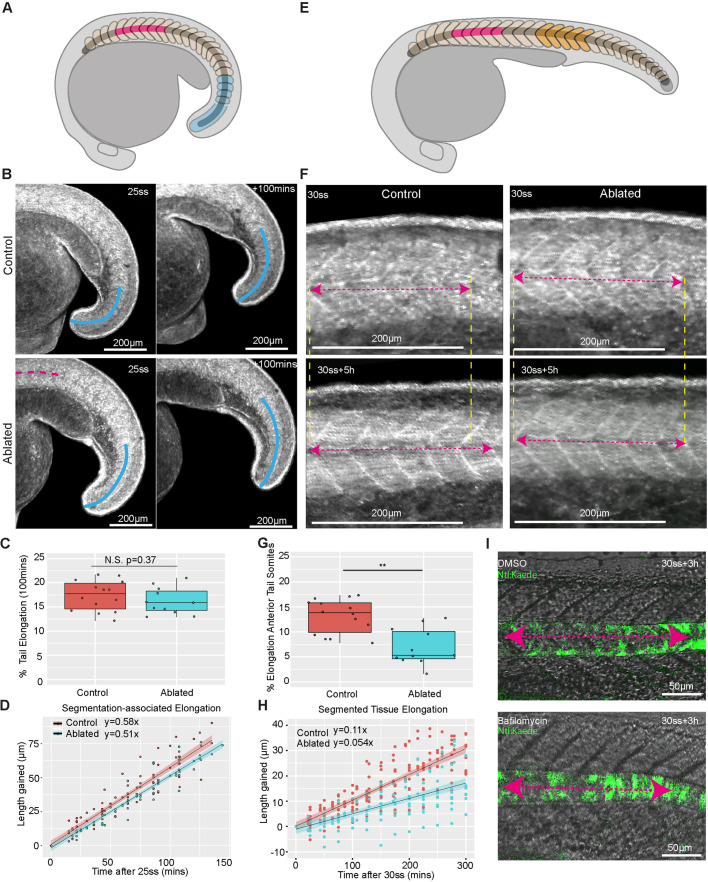
**Notochord cell expansion contributes to segmented tissue elongation during post-tailbud stages in zebrafish embryos.** (A) Schematic showing the ablated region (pink) and region tracked for measuring segmentation-associated elongation (blue) in the developing embryo. (B) Control and ablated LIFEACT-GFP-expressing embryos imaged at the 25ss and 100 min later. Overlaid blue curves show the region tracked for elongation measurements. The pink line indicates the region where the notochord was ablated. (C) Percentage elongation of the tail region in control and ablated embryos (*n*=14 control embryos, *n*=11 ablated embryos, *P*=0.37). N.S., not significant (Mann–Whitney U test). (D) Length gained in the posterior tail of tailbud-stage control and ablated embryos over time. (E) Schematic showing the ablated region (pink) and region tracked for measuring segmented tissue elongation (orange). (F) Elongation of a five-somite region in control and ablated post-tailbud stage LIFEACT-GFP-expressing embryos. Pink dashed arrows indicate the length of the measured region and yellow dashed lines indicate the anterior and posterior extents of the measured region. (G) Percentage elongation of a five-somite region in control and ablated post-tailbud-stage embryos over a period of 5 h (*n*=14 control and *n*=11 ablated embryos; **P<0.001, Mann–Whitney U test). (H) Length gained in a five-somite region in control and ablated post-tailbud-stage embryos over time. (I) Length of a five-somite region in representative DMSO and bafilomycin-treated post-tailbud-stage embryos. Pink dashed arrows indicate the length of the measured region.

We observed that elongation during tailbud stages was associated with the segmentation of posterior unsegmented tissue. To investigate whether notochord cell expansion impacted this mode of elongation, we performed site-specific ablations of anterior notochord cells at the onset of vacuolation and measured posterior tail elongation during tailbud stages of development (Fig. 3A,B). Posterior tail elongation was not significantly affected in ablated embryos (Fig. 3C,D). Strikingly, segmentation-associated elongation was able to continue in an embryo with an ablation extending into the posterior notochord (Movie 5), and presomitic mesoderm progenitors continued to exit the tailbud in ablated embryos (Fig. S4A). Together, these results demonstrate that segmentation-associated elongation is a robust process that can continue in the absence of notochord cell expansion.

To investigate whether notochord cell expansion impacts the segmented tissue elongation observed during post-tailbud stages, we measured the length of a five-somite region from 30ss over a 5 h period in embryos with and without anterior notochord ablations. The area used for quantifying segmented tissue elongation was located in an equivalent region along the axis in control and ablated embryos and positioned posteriorly away from the site of notochord ablation in the ablated case (Fig. 3E). As characterised above, this region was adjacent to either posteriorly directed notochord cell movement (control) or anteriorly directed notochord cell movement (ablated). Segmented tissue elongation was approximately halved in ablated embryos (Fig. 3F-H; Movie 6); inhibiting vacuolation with bafilomycin (Ellis et al., 2013) had an even greater effect, blocking elongation of segmented tissue (Fig. 3I; Fig. S4B-D). Together, these results reveal a role for notochord cell expansion in elongating segmented tissue in post-tailbud-stage embryos.

### Notochord progenitors provide a source of resistance to anterior notochord cell expansion that facilitates the elongation of segmented tissue

Unexpanded notochord cells in the posterior exhibited less movement relative to adjacent tissues compared with anterior expanded cells, suggesting that they may resist the force generated by cell expansion. Therefore, we hypothesised that expanding vacuolated notochord cells push against unexpanded posterior notochord cells, generating a stress along the axis that contributes to segmented tissue elongation. We first determined whether the addition of unexpanded cells to the posterior notochord was coordinated with the progression of notochord cell expansion posteriorly along the axis. Using *noto* expression to mark the notochord progenitor domain, we found that notochord progenitor domain volume decreased as development progressed (Fig. S5A,C), corresponding to a decrease in the length contributed to the posterior end of the notochord by progenitors (Fig. S5B,D). Next, we attempted to prevent the contribution of cells to the posterior notochord by ablating the notochord progenitor population (Fig. S5E). Although axis elongation defects occurred (Fig. S5F), the notochord progenitor pool was able to recover after ablation and re-form the posterior notochord (Fig. S5H,I). Intriguingly, elongation resulting from the segmentation of the final few somites in the region where the posterior notochord had been recovered was unaffected (Fig. S5G,J). These findings highlight the robustness of posterior processes that contribute to axis elongation, in terms of both cell contribution to the posterior notochord and segmentation-associated elongation.

**Fig. 4.**
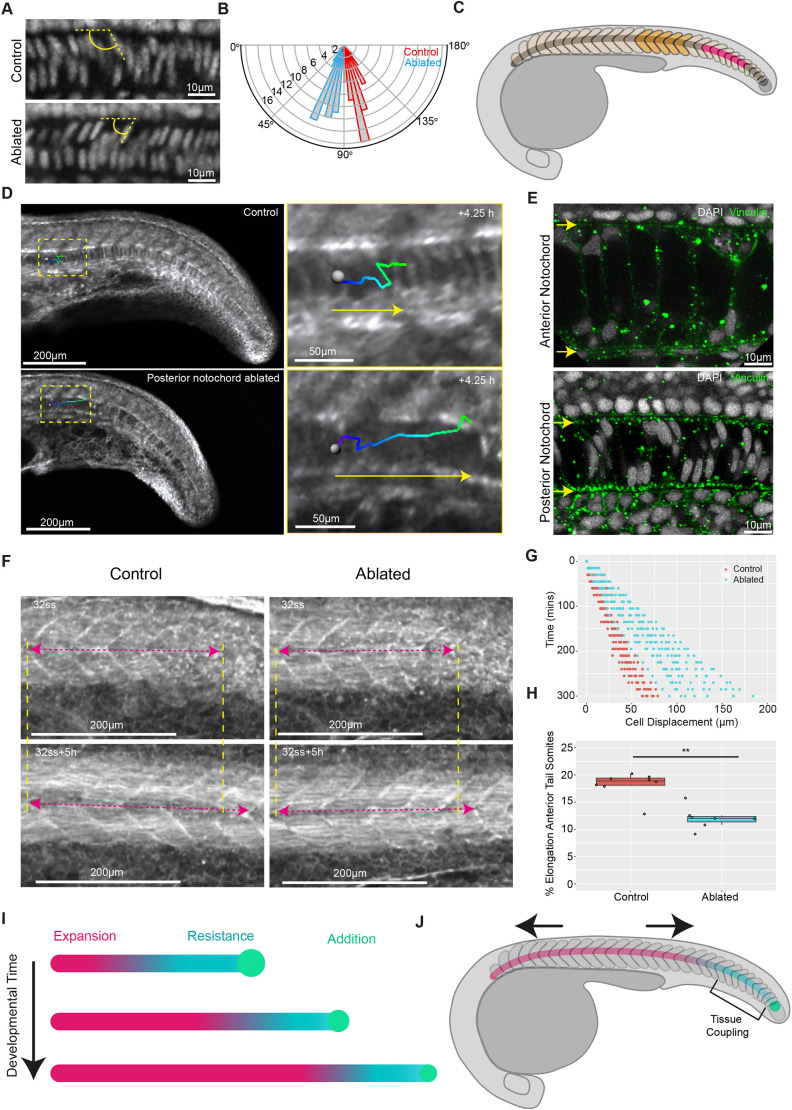
**Notochord progenitors provide a source of resistance to notochord cell expansion, facilitating segmented tissue elongation in zebrafish embryos.** (A) DAPI-stained notochord nuclei located in the anterior tail region in fixed control and ablated embryos. The angle between dorsally located nuclei and the notochord AP axis is indicated in yellow. (B) Polar histogram showing the distribution of angles between dorsally located nuclei and the notochord AP axis in control and ablated embryos (*n*=12 embryos per condition, five angles measured per embryo; *P*<0.001). (C) Schematic showing the ablated posterior unexpanded region (pink) and region used for measuring segmented-tissue elongation (orange). (D) Manual tracking (green-blue lines) of expanding notochord cells in a representative control and posteriorly-ablated embryo. Yellow arrows indicate direction of cell movement. (E) Vinculin and DAPI immunostaining in representative anterior and posterior notochord regions. Yellow arrows mark the dorsal (top) and ventral (bottom) extents of the notochord. (F) Elongation of a five-somite region in control and posteriorly ablated post-tailbud-stage embryos. The yellow dashed lines indicate the anterior and posterior extent of measured regions and the pink dashed arrows indicate the length of the measured region. (G) Notochord cell displacement over time in control and posteriorly ablated embryos (*n*=4 and *n*=5 embryos, respectively, 3 tracks per embryo). (H) Percentage elongation of a five-somite region in control and posteriorly ablated embryos (*n*=8 and *n*=7 embryos, respectively; **P<0.01, Mann–Whitney U test). (I) Notochord cell expansion progresses posteriorly along the axis. Expanding cells (pink) push against resisting unexpanded notochord cells (blue) added by posterior notochord progenitors (green), generating a stress along the notochord. (J) The somitic compartment anterior to the posterior resistance point undergoes an AP stretch (indicated by the black arrows). Tissue coupling between notochord cells and the somitic compartment is proposed to occur in the posterior of the embryo.

Given that the notochord re-formed after notochord progenitor ablation, we focussed our perturbations on the already-formed posterior notochord. In embryos entering post-tailbud stages of development, notochord cell expansion had progressed posteriorly along the axis to the anterior tail region (close to the end of the yolk extension), where it meets the unexpanded notochord cells (Fig. S6C). To investigate whether unexpanded notochord cells are subject to a force generated by anterior notochord cell expansion, we imaged the nuclei of unexpanded notochord cells in this region in control embryos, and immediately posterior to an ablation site in ablated embryos (Fig. 4A). Nuclei in control embryos were angled towards the posterior, whereas nuclei posterior to an ablation site were angled towards the anterior (Fig. 4B; Fig. S6A), suggesting that unexpanded notochord cells are being deformed by a posteriorly directed force arising from notochord cell expansion.

To investigate whether unexpanded posterior notochord cells provided a source of resistance to notochord cell expansion, we ablated these cells in post-tailbud-stage embryos and manually tracked expanding notochord cells immediately anterior to the ablation site (Fig. 4C,D; Fig. S6C). The posterior displacement of expanding cells was increased in ablated embryos compared with controls (Fig. 4G; Movie 7), suggesting that posterior notochord cells do provide a source of resistance. We investigated notochord cell adhesion to the sheath surrounding the notochord as a potential source of this resistance. Visualising the cell-extracellular matrix (ECM) adhesion protein vinculin revealed that vinculin puncta were located at the interface between notochord cells and the surrounding sheath in the posterior, whereas far fewer puncta were observed at this interface in the anterior, where notochord cells are expanded (Fig. 4E; Fig. S6B). Finally, we found that elongation of somites located anterior to the posterior notochord ablation site (Fig. 4C) was decreased (Fig. 4F,H). These findings suggest that posterior unexpanded notochord cells provide a source of resistance to notochord cell expansion that facilitates the elongation of segmented tissue in post-tailbud-stage embryos.

Our findings show that, although segmentation-associated axis elongation can progress independently of notochord cell expansion, this expansion plays a key role in elongating segmented tissue during later stages of embryo development, in agreement with phenotypes observed in embryos with defective notochord vacuolation (Sun et al., 2020; Bagwell et al., 2020). Physical coupling between notochord cells and the somitic compartment is thought to be stronger in the posterior versus the anterior of the embryo (Dray et al., 2013; Tlili et al., 2019); the resulting impact of this is observed in the larger displacement between these tissues in anterior regions where notochord cells have expanded and smaller displacement in posterior regions where expansion is yet to occur (Fig. 2A-C). Physical coupling is likely facilitated by a shared ECM interface between the notochord and somitic compartment (Guillon et al., 2020; Dray et al., 2013), with more vinculin puncta present at this interface in the posterior and fewer in the anterior (Fig. 4E). Therefore, we propose a model in which the progression of vacuolation posteriorly along the notochord leads to the posterior displacement of expanded (vacuolated) notochord cells, which is resisted by unexpanded cells in the posterior undifferentiated notochord (Fig. 4I). As expanding notochord cells push on the posterior notochord, coupling between the notochord and somitic compartment in the posterior would facilitate stress transmission to the segmented tissue located anterior to the coupled region (Fig. 4J). Over time, the posterior notochord region resisting the push from expanding notochord cells will shrink, as vacuolation progresses along the notochord and notochord progenitors are depleted (Fig. 4I), facilitating stress transmission to increasingly posterior regions of the somitic compartment as development progresses.

In summary, we highlight the importance of considering multi-tissue mechanical interactions in mechanisms of embryo axis elongation and report a role for notochord cell expansion in mediating the elongation of somites. Further studies investigating whether force generated by notochord cell expansion acts to stretch myofibres directly, or impacts myofibre differentiation and elongation via indirect mechanochemical regulation, will deepen our understanding of these multi-tissue interactions.

## MATERIALS AND METHODS

### Zebrafish strains and maintenance

This research was regulated under the Animals (Scientific Procedures) Act 1986 Amendment Regulations 2012 following ethical review by the University of Cambridge Animal Welfare and Ethical Review Body (AWERB).

Adult zebrafish (*Danio rerio*) and embryos were reared at 28°C and staged using the number of somites. Wild-type embryos used in this study were of the TL strain. Prior to live imaging, embryos were anaesthetised with tricaine (MS-222; Sigma-Aldrich).

The following zebrafish lines were used in this study: H2B-GFP, Tg(actb2:LIFEACT-GFP) (hereafter ‘LIFEACT-GFP’) and pNtl:Kaede^16^ (kind gift of Ben Martin, Stony Brook University, USA).

Tailbud stages correspond to the stages during the segmentation period after a tailbud has formed, whereas post-tailbud stages correspond to the pharyngula period, when the tailbud has started to degenerate (Kimmel et al., 1995).

### Time-lapse live imaging

Embryos were mounted in glass-bottomed dishes (MatTek) with the head and yolk submerged in 3% methylcellulose (Sigma-Aldrich) to hold them still, while allowing movement of the tail and ensuring embryos could continue to develop normally. Embryos were then submerged in E3 medium (5 mM NaCl, 0.17 mM KCl, 0.33 mM CaCl_2_, 0.33 mM MgSO_4_, 5-10% Methylene Blue) and an eyelash tool was used to reposition them in the methylcellulose so that they were lying flat on the bottom of the dish. Dishes with mounted embryos were transferred to a Zeiss LSM 700 confocal microscope equipped with a heated chamber set to 28°C. A 10× air objective (NA=0.45) was used to capture the whole body of developing embryos for time-lapse movies.

### Drug treatments

Bafilomycin A1 (an inhibitor of vacuolar type H^+^-ATPases required for vacuole expansion; Sigma-Aldrich) was used to inhibit vacuolation in the notochord of the developing zebrafish embryos. Embryos (16ss) were submerged in E3 medium with bafilomycin at a final concentration of 0.5 μM. Embryos were then incubated for 6 h at 28°C. Vacuolation in the notochord of LIFEACT-GFP embryos was imaged live using a 20× air objective using a Zeiss LSM 700 confocal microscope. Live imaging of somite elongation in DMSO- and bafilomycin-treated embryos was performed using pNtl:Kaede^16^-expressing embryos pretreated with 0.25 µM bafilomycin (or DMSO of an equivalent volume) and imaged from the 30ss onwards in a multi-well chamber using a Zeiss LSM 700 confocal microscope.

### Photolabelling

Wild-type zebrafish embryos were injected with ∼200 pg of nuclear-targeted Kikume (NLS-KikGR) mRNA recovered from *Escherichia coli* plasmid stocks (a kind gift of Ben Martin, Stony Brook University, USA) at the one-cell stage and incubated in the dark. Embryos were mounted in glass-bottomed dishes and transferred to a Zeiss LSM 700 confocal microscope as described above for live imaging. To investigate the movement of notochord cells relative to adjacent regions of the somitic compartment and neural tube, rectangular stripes were defined at different points along the embryo. The distance between the anterior extent of the stripes defined in each tissue was used to measure the relative shift in notochord cell movement (Fig. S3). Photoconversion of NLS-KikGR was performed using a Zeiss LSM 700 confocal microscope with a 20× air objective and scanning the 405 nm laser at 11% power within defined regions. Photolabelling was confirmed by simultaneously visualising both NLS-KikGR emissions using 488 nm and 561 nm lasers.

### Cell tracking

The centre of notochord cells in LIFEACT-GFP-expressing embryos was manually tracked using Imaris cell imaging software relative to a nearby somite boundary located in an equivalent region of the axis in each embryo. Reference frames were generated using the ‘reference frame’ tool in Imaris, the *x*-axis was aligned with the AP axis and the origin was placed at the somite boundary.

### Hybridisation chain reaction

Hybridisation chain reactions (HCR) was used to visualise gene expression in fixed embryos following the method described in detail by Choi et al. (2014). Embryos were fixed using 4% (w/v) paraformaldehyde in DEPC-treated PBS. HCR probes (2 pmol; Molecular Technologies, 2684/B887) were hybridized at 37°C overnight in 500 μl 30% formamide hybridisation buffer. This was followed by repeated washing at 37°C using 30% formamide probe wash buffer. Probes were detected by annealing of fluorescent hairpins in amplification buffer overnight at room temperature followed by repeated washing in 5× SSC 0.001% Tween 20. Samples were finally counterstained using DAPI. DNA probes, fluorescent hairpins and buffers were purchased from Molecular Instruments. The notochord progenitor domain was visualised using probes targeted to *noto* (also known as *flh*). The extent of ablation damage was assessed using probes to visualise *shh* expression.

### Immunohistochemistry

Embryos were fixed in 4% paraformaldehyde overnight at 4°C. They were then washed and blocked in 4% goat serum in PBDT [PBS, DMSO and 0.1% Triton X-100 (Sigma-Aldrich)] before being incubated overnight at 4°C with anti-vinculin primary antibody (ab91459, Abcam) at 1:200 in 4% goat serum in PBDT. Embryos were washed in PBDT and incubated with secondary antibodies (Alexa Fluor 488-conjugated goat anti-rabbit IgG, A11034, Thermo Fisher Scientific) overnight at 1:500 in 4% goat serum in PBDT.

### Photoablation

Laser ablation was performed using a TriM Scope II Upright 2-photon scanning fluorescence microscope equipped with a tunable near-infrared laser. InspectorPro software was used to control the microscope and a laser power of ∼1.3 W was used for ablation.

Notochord ablations were performed in the plane of the notochord using the LIFEACT-GFP reporter line to visualise notochord cells. Notochord cells were ablated in either the anterior or posterior regions of the notochord in embryos at 16-18ss to ensure that cells fated to become the vacuolated cell type were destroyed before they had drawn in water and osmotically expanded. Ablations may have included some undifferentiated sheath-fated cells (Yamamoto et al., 2010).

For notochord progenitor ablations, the morphology of the notochord progenitor cell population was used to determine the location of the notochord progenitors. Ablations were carried out to ensure as many of the notochord progenitors as possible were ablated, without ablating any other progenitor types.

### Image processing and analysis

Images were processed either in ImageJ/Fiji or Imaris.

### Length measurements

For length measurements, the ImageJ plug-in ‘Kappa’ was used. NLS-KikGR-, H2B-GFP- and LIFEACT-GFP-expressing embryos were used to track the elongation arising from segmented and unsegmented regions of the axis over time. A maximum projection of the nuclear-GFP or actin-GFP channel was used so that somite boundaries could be visualised and used to define the segmented and unsegmented regions. Embryo side views were used to measure the length of regions of the axis. The elongation of each region was measured using points to generate a spline, and the points were adjusted so that a smooth curve fitting the curvature of the region passed through the midpoint of somites in that region.

### Notochord progenitor domain volume

To measure the volume of the notochord progenitor domain at different developmental stages, *noto* expression was visualised using HCR (see above) and acquisition of confocal *z*-stacks of fixed 20ss, 25ss and 30ss embryo tails. These images were imported into Imaris, allowing for visualisation in 3D. The ‘surfaces’ function in Imaris was used to generate a surface around the *noto* expression signal, with surface volume given as an output.

### Vacuole AP length measurements

The ‘oblique slicer’ tool in Imaris was used to find a plane that passed through the middle of the notochord, where the maximum length of vacuoles could be accurately visualised. The ‘measurements’ tool was used to make two points per vacuole defining a line passing through the maximum AP length of the vacuole. Line lengths were calculated using an inbuilt function in Imaris. Vacuole lengths were measured in wild-type or DMSO-treated LIFEACT-GFP-expressing embryos.

### Nuclei angle measurements

The ‘measurements’ tool in Imaris was used to measure the angle between nuclei located dorsally in the notochord and in the dorsal AP edge of the notochord in fixed DAPI-stained embryos with and without notochord ablations.

### Data analysis

Data were stored in Excel (Microsoft) spreadsheets and analyses were performed using the programming language ‘Python’. All data plots were generated in Python using the ggplot library. Box plots show the median, upper and lower quartiles, and whiskers represent 1.5 times the interquartile distance. Linear regression analysis was used to fit a line to data in scatter plots showing length gained versus time. Scatter plots were generated using the geom_point function. The scikit-learn module was used for linear regression analysis. All data points are shown and included in statistical analyses.

### Statistical analysis

The Mann–Whitney U nonparametric statistical test was used to test whether two independent samples came from populations with the same distribution. This test was implemented in Python using the scipy.stats.mannwhitneyu() function.

The Kruskal–Wallis nonparametric statistical test was used to test whether more than two independent samples came from populations with the same distribution.

Pairwise comparisons between samples were performed in a post-hoc fashion to identify whether compared samples came from populations with the same distribution. The Kruskal–Wallis test was implemented using the stats.kruskal() function, and the post-hoc test was implemented with the sp.posthoc_conover() function. In the case of segmentation-associated elongation in anterior-ablated and control embryos, a power analysis was conducted to estimate sample sizes needed to detect a difference of 3% between the means of each group at a standard power score of 0.8.

In all cases, *P*<0.05 was taken as a threshold for significance.

## Supplementary Material

Supplementary information

Reviewer comments
