# Supplementary material for: Anterior expansion and posterior addition to the notochord mechanically coordinate zebrafish embryo axis elongation
Source: Development. 2021 Jul 21;148(18):dev199459. doi: 10.1242/dev.199459 (PMC8327291; doi:10.1242/dev.199459)
Supplement: Supplementary information [file develop-148-199459-s1.pdf]

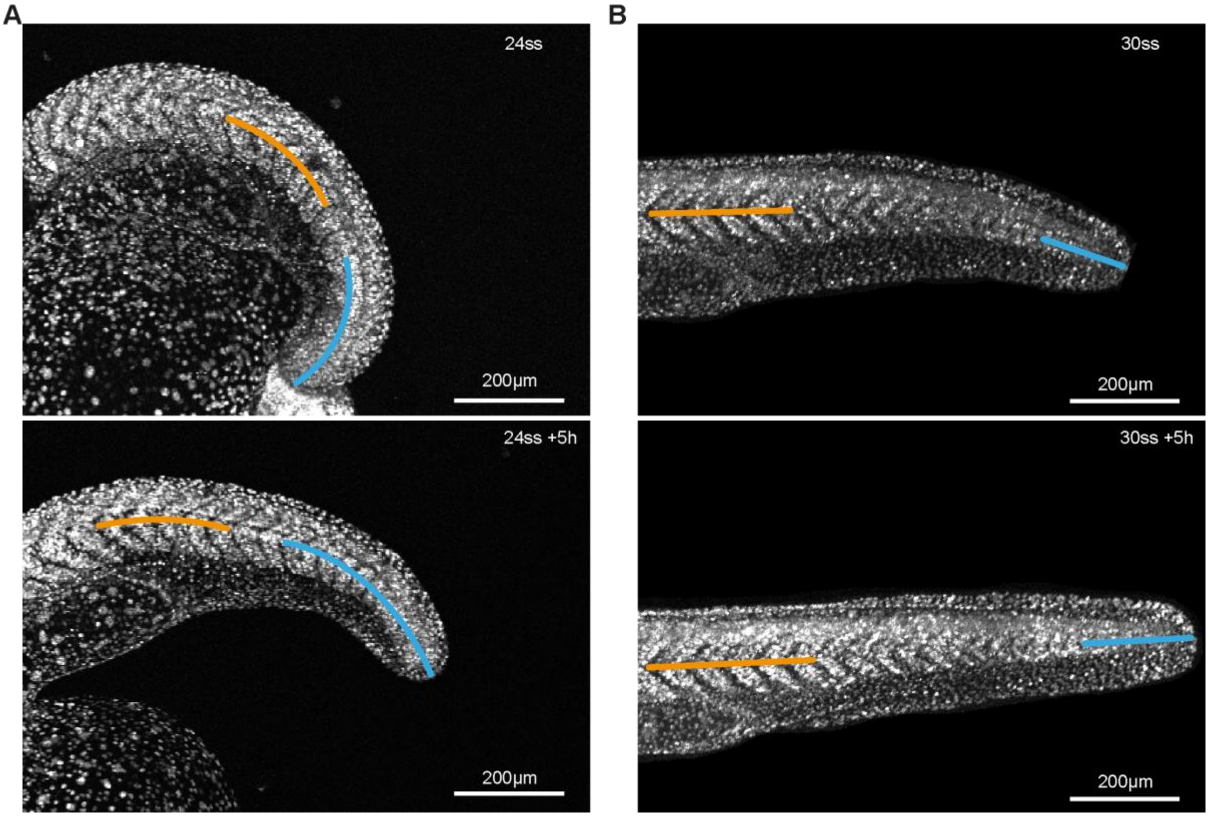

Fig. S1. Segmentation-associated and segmented tissue elongation during tailbud and post-tailbud stages of development.

(A) A live zebrafish embryo expressing NLS-KikGR at the 24-somite stage and 5 hours later.

(B) A live zebrafish embryo expressing NLS-KikGR at approximately the 30-somite stage and 5 hours later.

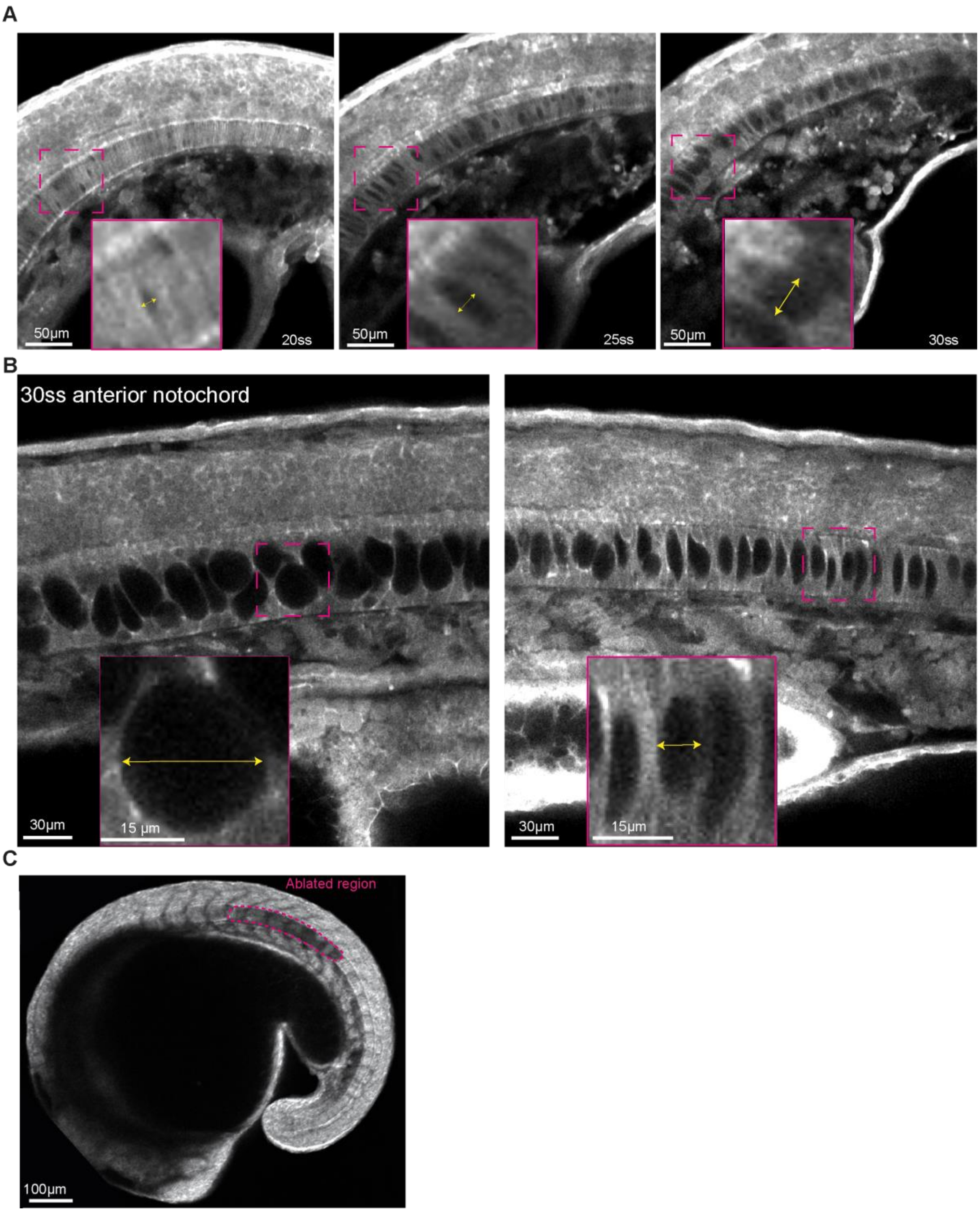

Fig. S2. Notochord cell expansion progresses temporally and spatially along the axis.

(A) Notochord cells in an equivalent region of a Lifeact-GFP expressing embryo at 20, 25, and 30-somite stages. Yellow arrows indicate direction in which length measurements were taken.

(B) Notochord cells in the anterior and posterior of a Lifeact_GFP expressing embryo. Yellow arrows indicate direction in which length measurement was taken.

(C) An anterior notochord ablation in a lifeact-GFP expressing embryo near the onset of vacuolation. Embryos were ablated at 18 somite stage.

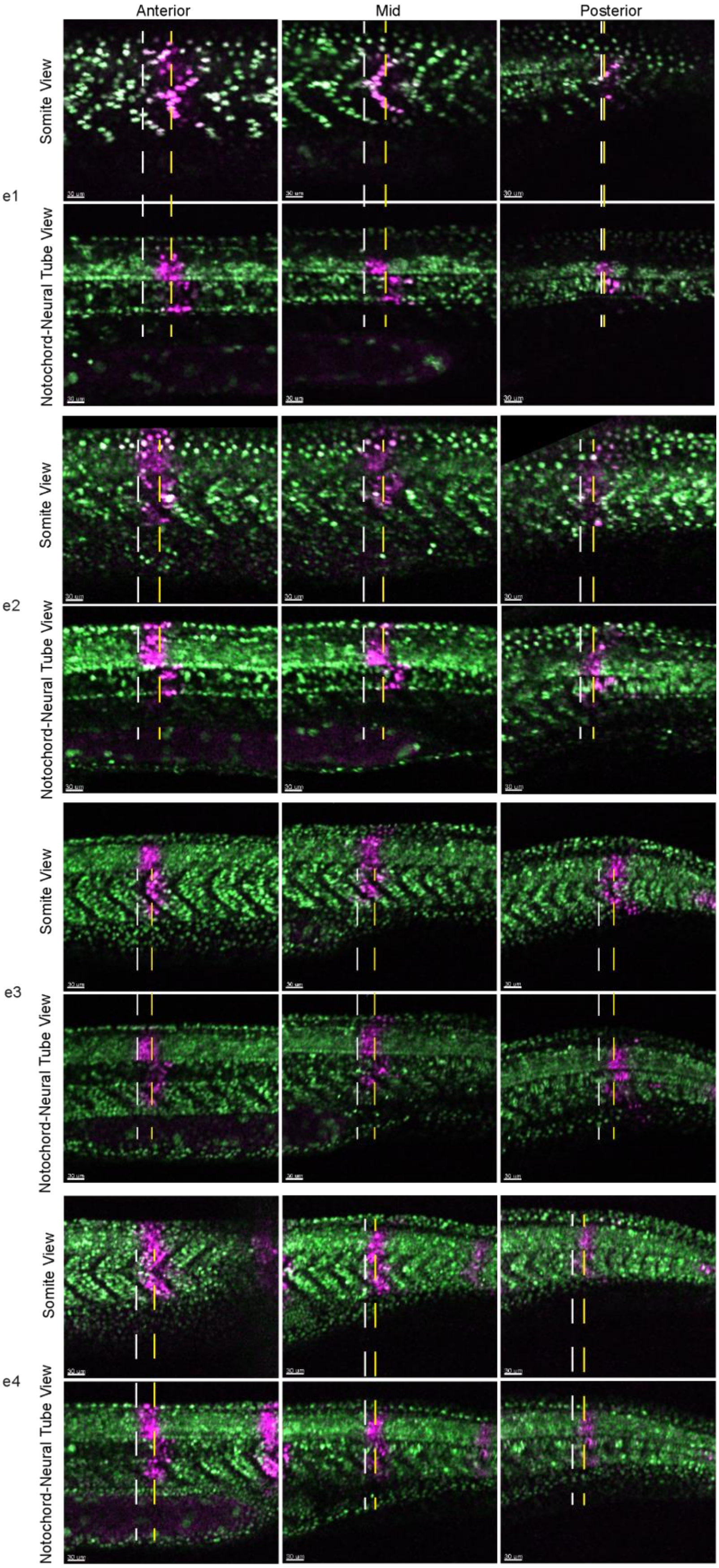

Fig. S3. Notochord and somite label at different regions along the axis in post-tailbud stage embryos. Images showing the anterior extent of notochord (yellow-line) and somite (white-line) photolabels used to measure the shift between tissues over a 5-hour period.

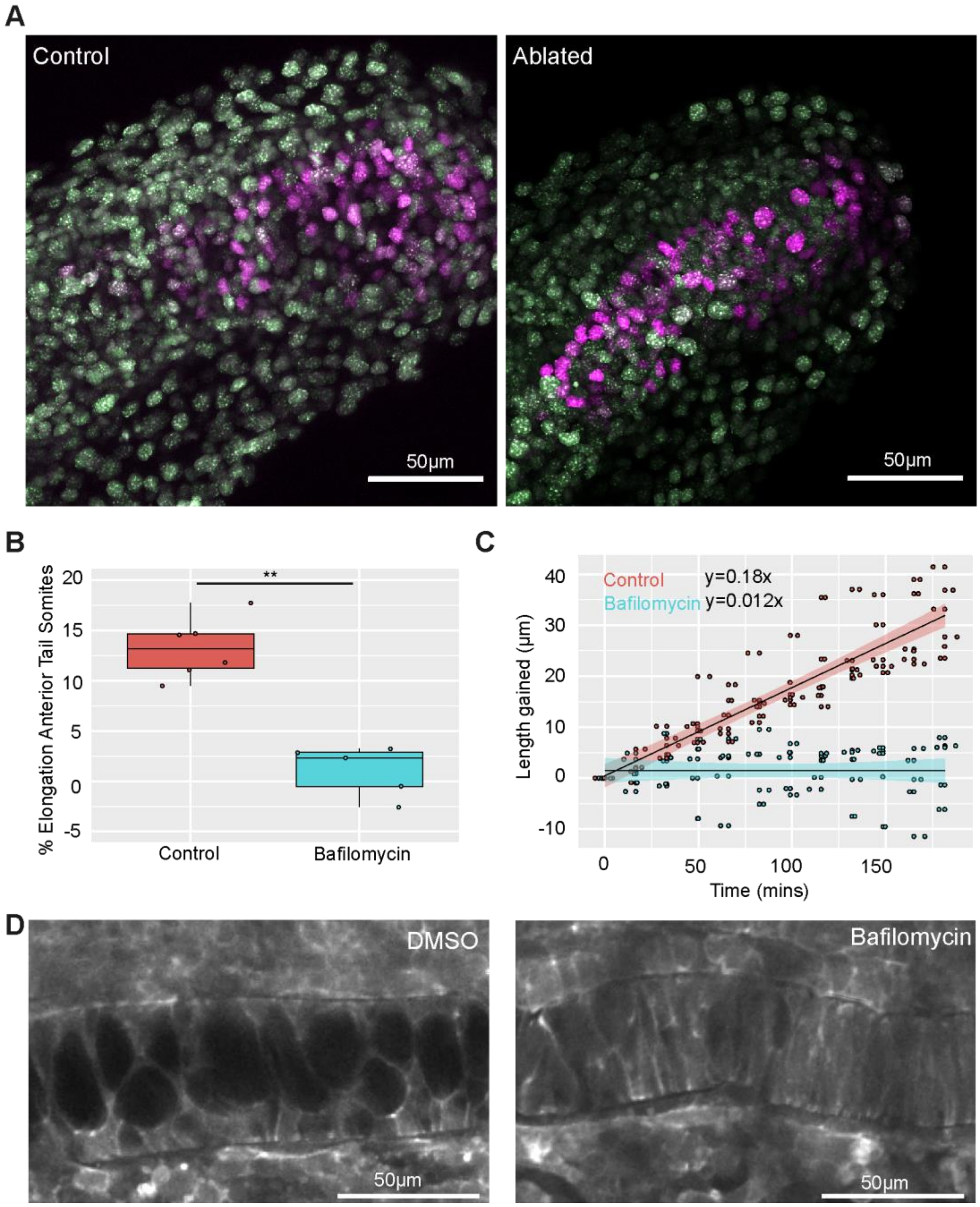

Fig. S4. Disrupted notochord cell expansion impacts segmented tissue-associated, not unsegmented tissue-associated elongation.

(A) Photolabels of presomitic mesoderm progenitors in control and ablated embryos imaged 4 hours after the time of labelling.(n=4 control and n=5 ablated embryos).
(B) Percentage elongation of a 5-somite region over a 3-hour period in DMSO and Bafilomycin treated post-tailbud stage embryos (n=6 control and n=5 Bafilomycin treated embryos, $p<0.01$).
(C) Length gained in a 5-somite region over time in DMSO and Bafilomycin treated embryos (n=6 control and n=5 Bafilomycin treated embryos).
(D) Images of the notochord in DMSO and Bafilomycin treated Lifeact-GFP expressing embryos.

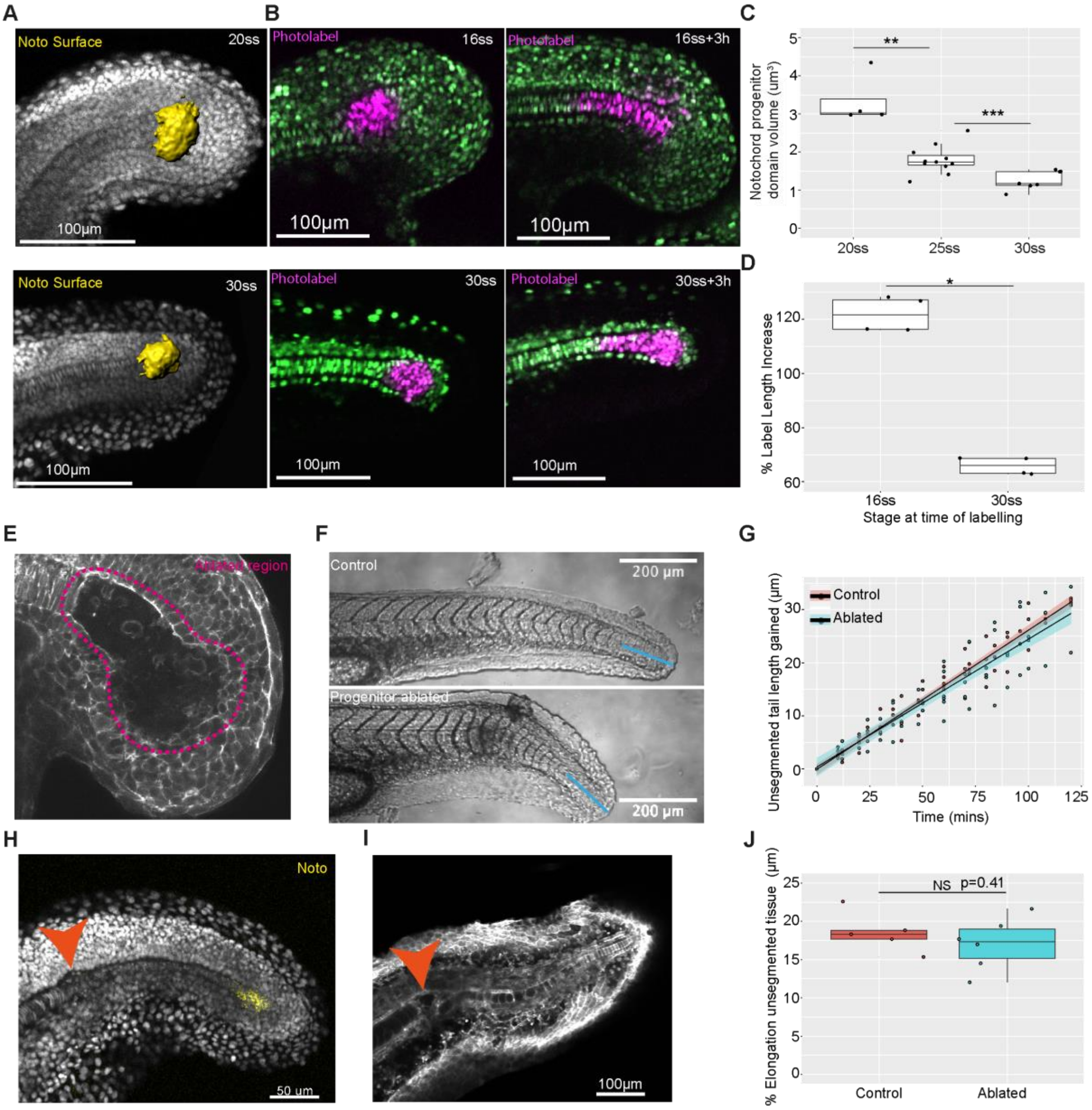

Fig. S5. Notochord progenitors recover after ablation and re-form the posterior notochord.

(A) 3D surfaces of the notochord progenitor domain generated using *Noto* gene expression in 20 and 30 somite stage embryos.
(B) Notochord progenitor photolabels in 16 and 30 somite stage embryos with corresponding images taken 3 hours after labelling.
(C) Notochord progenitor domain volume in 20, 25 and 30 somite stage embryos (n=5, n=13, n=7 embryos respectively, p<0.01, p<0.001 respectively).
(D) Photolabel AP length increase over 3 hours in 16 and 30 somite stage embryos (n=4 embryos per stage, p<0.05).
(E) Representative image of a 16-somite stage embryo with notochord progenitors ablated.
(F) Representative images of control and progenitor ablated post-tailbud stage embryos. The blue line indicates the region of residual unsegmented tissue tracked over time.
(G) Length gain in a small region of unsegmented tissue over time in control and progenitor ablated post-tailbud stage embryos (n=6 control and n=7 ablated embryos).
(H) Representative image of an embryo fixed approximately 5 hours after notochord progenitor ablation. The gene expression marker ‘*Noto’* marks the notochord progenitors.
(I) Representative image of an embryo imaged approximately 24 hours after notochord progenitor ablation. The recovered notochord is visualised using a lifeact-GFP reporter.
(J) Percentage elongation of unsegmented tissue in the tail region after recovery from notochord progenitor ablation over approximately 100 minutes (n=5 control and n=6 ablated embryos, p=0.41).
Orange arrows indicate the internalised ablated tissue.

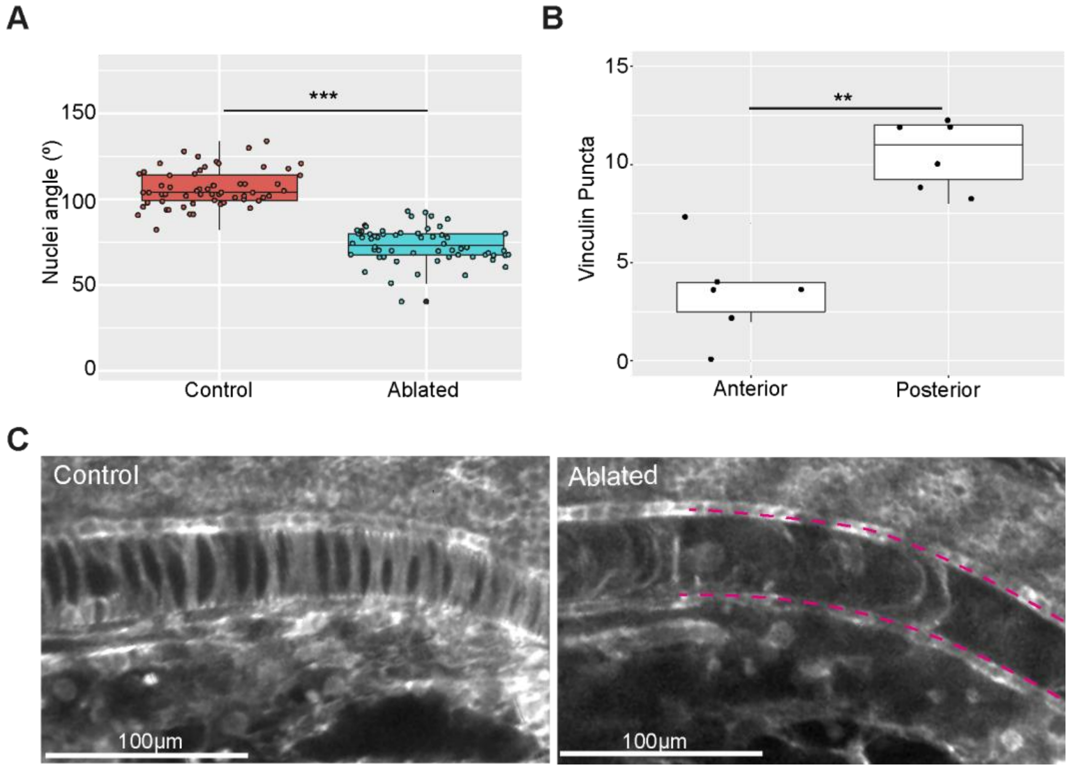

Fig. S6. Unexpanded posterior notochord cells resist anterior cell expansion.

(A) Angles between dorsally located nuclei and the notochord AP axis (º) in control and ablated embryos (n=12 embryos per condition, 5 angles measured per embryo, p<0.001).
(B) The number of vinculin puncta present at a notochord-ECM sheath interface in the anterior and posterior (n=3 embryos, 2 interfaces measured per embryo).
(C) Notochord cells located close to the end of the yolk extension in Lifeact-GFP expressing control and posteriorly-ablated embryos (magenta lines).

Development • Supplementary information

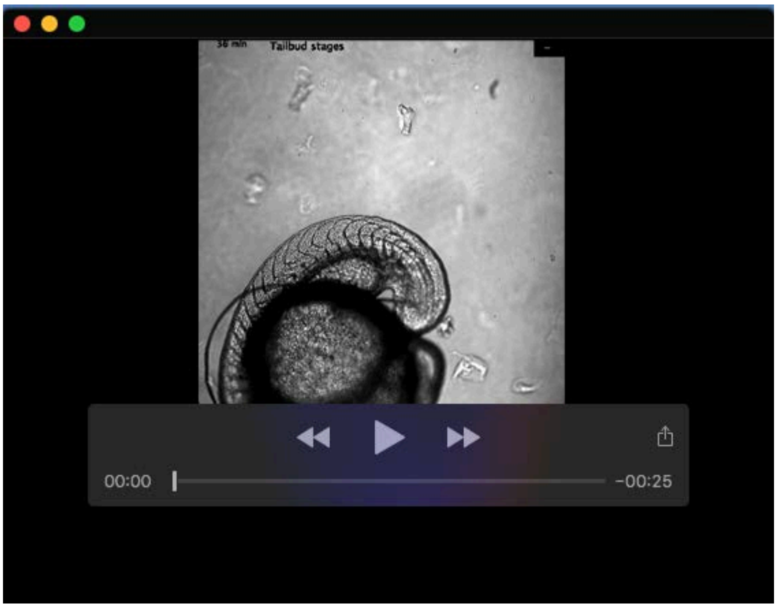

Movie 1. Tailbud and post-tailbud stages of development. A brightfield movie showing embryos transitioning from tailbud to post-tailbud stages of elongation. Time is in minutes.

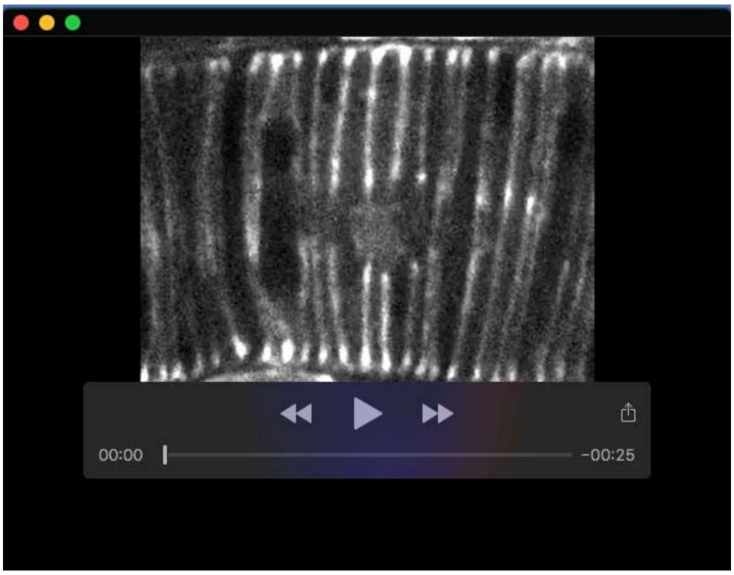

Movie 2. Notochord cell ablation. Multi-photon ablation of notochord cells in a lifeact-GFP expressing embryo.

Development • Supplementary information

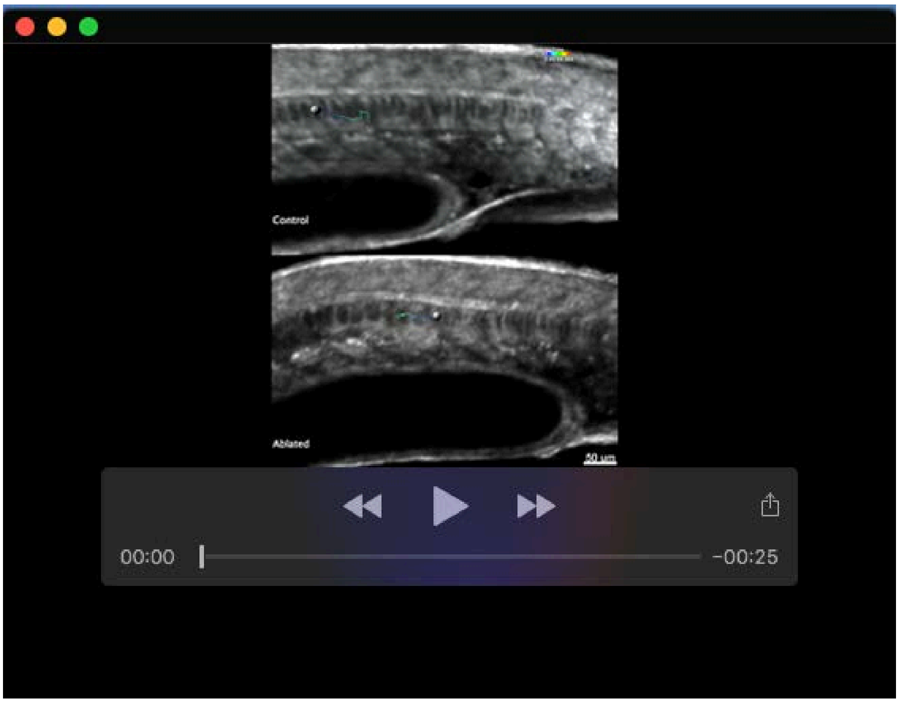

Movie 3. Vacuolated cell movement in control and anterior-ablated embryos. Movement of expanding vacuolated cells in a control embryo and an embryo with an anterior notochord ablation. Time is in hours.

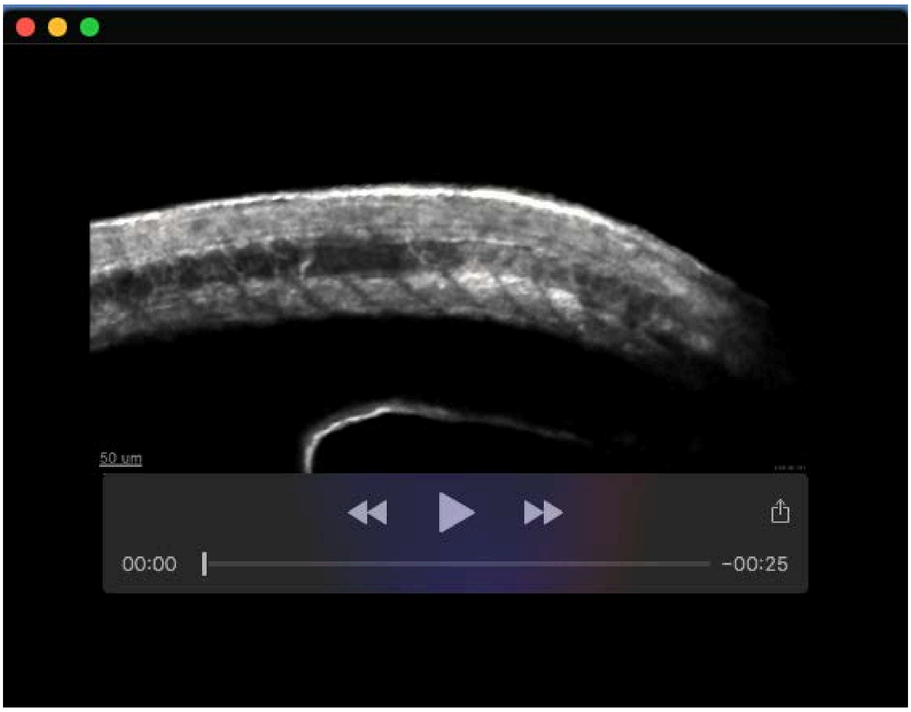

Movie 4. Vacuolated cells move into the ablated space. Movement of expanding vacuolated cells on either side of an anterior notochord ablation. Time is in hours.

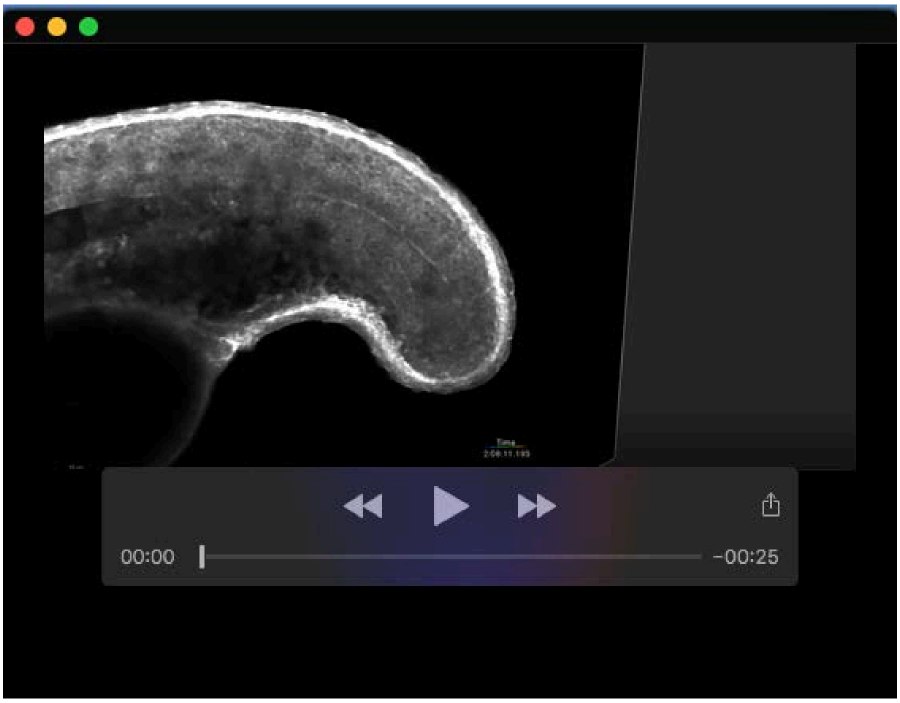

Movie 5. Segmentation-associated elongation is robust to notochord perturbation. Posterior tail elongation in an embryo with an ablation extending into the posterior notochord. Time is in hours.

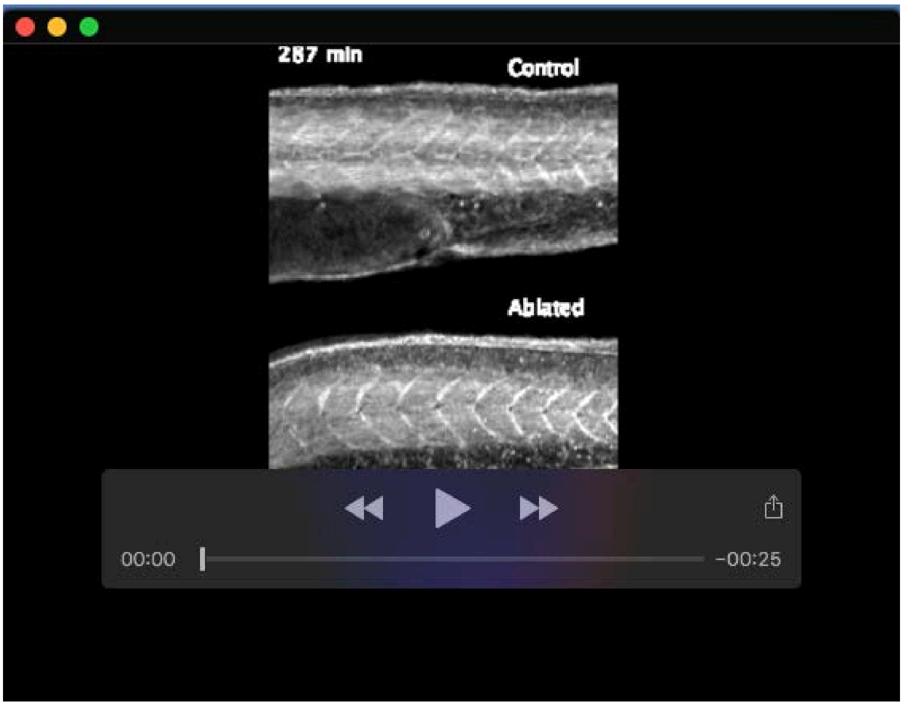

Movie 6. Segmented tissue elongation is decreased in embryos with disrupted notochord cell expansion. Elongation of a region of 5 somites in a control and ablated embryo. Time is in minutes.

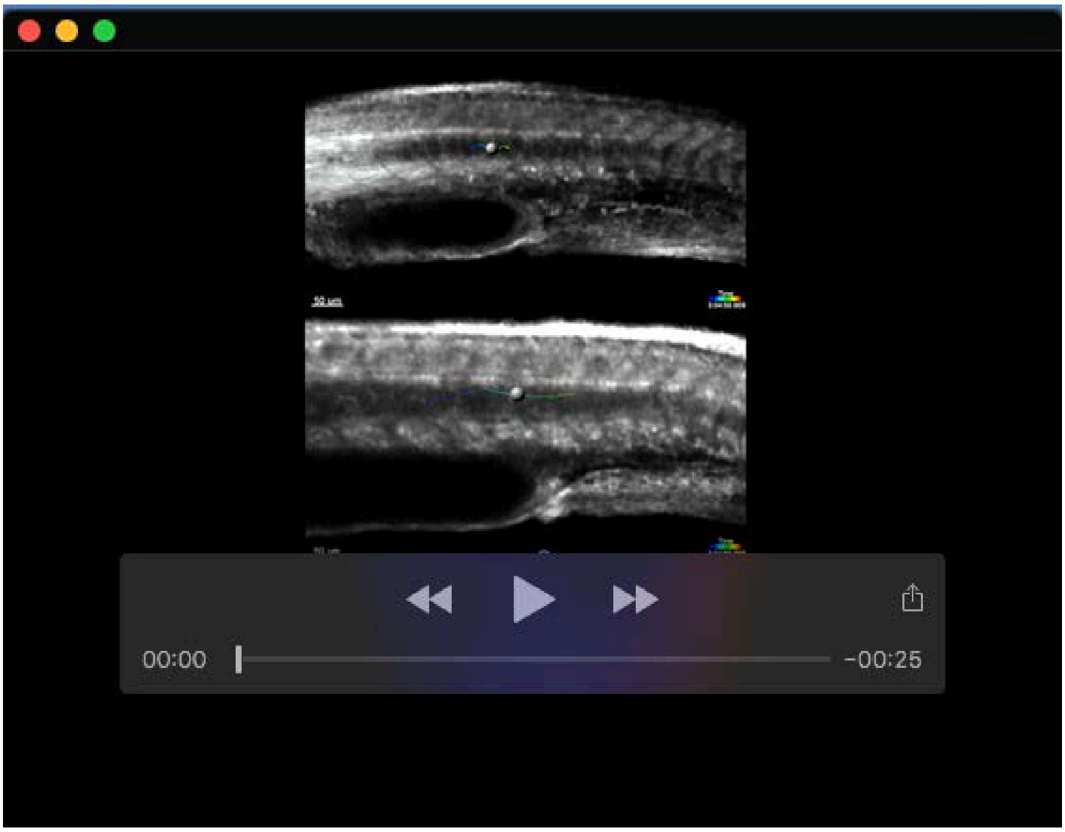

Movie 7. Vacuolated cells move faster when posterior unexpanded cells are removed. Movement of expanding vacuolated cells in a control embryo and an embryo with a posterior notochord ablation. Time is in hours.
