# Supplementary material for: Anterior expansion and posterior addition to the notochord mechanically coordinate zebrafish embryo axis elongation
Source: Development. 2021 Jul 21;148(18):dev199459. doi: 10.1242/dev.199459 (PMC8327291; doi:10.1242/dev.199459)
Supplement: Reviewer comments [file develop-148-199459_review_history.pdf]

Anterior expansion and posterior addition to the notochord mechanically coordinate zebrafish embryo axis elongation

Susannah B. P. McLaren and Benjamin J. Steventon
DOI: 10.1242/dev.199459

Editor: James Briscoe

Review timeline
Original submission: 26 January 2021
Editorial decision: 22 February 2021
First revision received: 5 May 2021
Accepted: 26 May 2021

Original submission

First decision letter

MS ID#: DEVELOP/2021/199459

MS TITLE: Anterior expansion and posterior addition to the notochord mechanically coordinate embryo axis elongation

AUTHORS: Susannah B P McLaren and Benjamin J Steventon

I have now received all the referees' reports on the above manuscript, and have reached a decision. The referees' comments are appended below, or you can access them online: please go to BenchPress and click on the 'Manuscripts with Decisions' queue in the Author Area.

As you will see, the referees express considerable interest in your work, but have some significant criticisms and recommend a substantial revision of your manuscript before we can consider publication. The referees request clarification on a number of points and have several helpful suggestions to contextualised your study within the relevant literature of vertebrate body axis morphogenesis. Referee 3 also questions whether some of the experiments are statistically underpowered and I would be grateful if you address this comment. Experimentally, I think repeating the cell labelling experiment at different AP levels and developmental times might provide insight into the degree of tissue-tissue coupling and this would strengthen your study.

If you are able to revise the manuscript along the lines suggested, which may involve further experiments, I will be happy receive a revised version of the manuscript. Your revised paper will be re-reviewed by one or more of the original referees, and acceptance of your manuscript will depend on your addressing satisfactorily the reviewers' major concerns. Please also note that Development will normally permit only one round of major revision.

We are aware that you may be experiencing disruption to the normal running of your lab that make experimental revisions challenging. If it would be helpful, we encourage you to contact us to discuss your revision in greater detail. Please send us a point-by-point response indicating where you are able to address concerns raised (either experimentally or by changes to the text) and where you will not be able to do so within the normal timeframe of a revision. We will then provide further guidance. Please also note that we are happy to extend revision timeframes as necessary.

© 2021. Published by The Company of Biologists under the terms of the Creative Commons Attribution License (https://creativecommons.org/licenses/by/4.0/).

Please attend to all of the reviewers' comments and ensure that you clearly highlight all changes made in the revised manuscript. Please avoid using 'Tracked changes' in Word files as these are lost in PDF conversion. I should be grateful if you would also provide a point-by-point response detailing how you have dealt with the points raised by the reviewers in the 'Response to Reviewers' box. If you do not agree with any of their criticisms or suggestions please explain clearly why this is so.

Reviewer 1

Advance summary and potential significance to field

In the manuscript by McLaren and Steventon the authors investigated the role of the notochord in axis elongation in zebrafish. Using quantitative microscopy methods and cells ablation as main experimental tool they showed that vacuolated cell expansion at the anterior of the notochord, resisted by the undifferentiated posterior end contributes to axis elongation. While some of the main conclusions were previously reached qualitatively by other studies, their quantitative analyses add precision and suggest a mode and timing of tissue-tissue coupling during axis elongation that is novel. Particularly interesting are their conclusions about the role of the notochord in the expansion of segmented tissue as it shows an aspect of notochord-dependent axis elongation that has not previously been examined in any detail and was somewhat dismissed by other studies focused on posterior cell addition.

Comments for the author

Overall, this is an interesting and straightforward study that reaches some clear conclusions and opens some questions for future studies. However, there are technical limitations due to the lack of specific markers, a need to perform additional controls, address experimental conditions and perhaps reconsider some discussion points. Some technical details and figure labelling also need attention.

Major points:
1-The authors always refer to notochord cells throughout the manuscript and do not make distinction between vacuolated and sheath cells. Due to the absence of specific markers for each population they cannot always tell if they are following or targeting vacuolated cells, sheath cells or the undifferentiated tissue. For example, the relative displacement of notochord and somites, Fig.2EF, and the cells followed in 2G likely are vacuolated cells. In contrast, in the undifferentiated tissue it is not possible to distinguish both populations and the whole rod may be displaced by the expansion of vacuoles more anteriorly. Whenever appropriate vacuolated cells need to be mentioned specifically as the relative movement of vacuolated cells due to vacuole expansion is also relative to the sheath epithelium in the anterior portion of the notochord. If the authors suggest the whole tissue, including the sheath epithelium, is being displaced they would need to use specific markers and more detailed observations. For specific markers see Yamamoto et al. (2010, PMID 20573700); Garcia et al. (2017, PMID 28648824); Dale et al. (2011, PMID 21723274).

2-The labeling experiment in Fig.2EF is both relatively simple and compelling. Perhaps if it is repeated at different AP levels and developmental windows we may learn more about the degree of tissue-tissue coupling, which is discussed later in the manuscript.

3-In the experiments presented in figure 3 and S3 they authors show a role for vacuolated cell expansion in segmented tissue that is highly interesting. However, they also present data in figure S3 that suggest a role in axis straightening that needs to be discussed critically as mutants with vacuole fragmentation or conditions in which vacuolated cell number are reduced lead to significant shortening of the axis without clear defects in straightening. Therefore, it is possible that the relatively mild straightening differences they observed are due to tissue damage rather than a direct result of a loss of notochord stiffness.

4-Could the authors show controls for the extent of damage produced by the ablations? Controls for potential damage to other tissues are also needed.

© 2021. Published by The Company of Biologists under the terms of the Creative Commons Attribution License (https://creativecommons.org/licenses/by/4.0/).

5-Following ablation, cells (presumably vacuolated) appear to be displaced anteriorly (Fig.2G). Are cells filling the space of the ablated cells? How is this response interpreted?

6-In figure a higher n is would be best for nuclei angle measurements.

Minor points:
7-One aspect that may need to be considered further is the interpretation of the role of the unexpanded notochord in resisting the force from anterior expansion. Even at early larval stages expansion continues at the posterior end and this process has an impact in axis elongation that is apparent in mutants, e.g. cavin1b (see Garcia et al. 2017, PMID 28648824; and Lim et al. 2017, PMID28648821), in which vacuolated cell disruption occurs after hatching. The extrusion of vacuolated cells seen in Romero et al (2018) and also Norman et al. (2018, PMID30249771) reflects internal pressure held by the sheath that was severed rather than a loss of resistance from the unexpanded notochord. This also brings up the need to consider the development of hydrostatic pressure throughout the notochord as vacuoles expand within the sheath that couples the whole structure.

8-While the effect of vacuolated cell ablation is likely not confounded by regenerative responses during the short time frame of their experimental setup, it is worth considering whether expansion of neighboring intact vacuolated cells and changes in cell packing (see Norman et al., PMID 30249771 and Garcia et al. 2017, PMID 28648824) may dampen the effect of cell ablation.

9-Markers in figures S3 (ntl:kaede?) and S4 (HCR for flh?) and not described.

10-Some details about the photolabeling experiments would be useful.

Reviewer 2

Advance summary and potential significance to field

The notochord has long been known to have an important role in the morphogenesis of the AP axis, shown nicely in zebrafish with a variety of mutants that affect the formation of the notochord. The authors here study the role of the notochord in axis elongation during the somitogenesis stages, which has not been well characterized, using targeted laser ablation. They nicely show that a combination of the anterior notochord expanding through vacuolation and providing a posterior-directed force together with addition of cells from the posterior progenitors, drives the morphogenetic events.

The work is beautifully done and well presented, and provides a much more careful understanding of the role of the notochord in vertebrate morphogenesis. The comments are just some suggestions to improve the text.

Comments for the author

Page 2, para 2 It seems a bit surprising to me that the noto mutant is not mentioned since it clearly shows in zebrafish how the absence of a notochord affects early embryo morphogenesis. It also shows that the notochord is having effects even by 24 hpf (what the authors call the end of the tailbud stages as shown for example in Talbot, 1995, Fig 1), whereas this paper makes it seem like the notochord only begins to have effects on morphogenesis after this stage. I think it would help to briefly integrate the pre-24 hpf effects in the text.

Page 3, para 2 With regards to the statement about “boundaries of 5 formed somites” is that 5 somites picked randomly or the same 5 somites in all embryos (ie somites 1-5)? It would help to be a bit more specific.

Page 3, para 3 It might be a bit better to say “starting at” rather than “during”. I also think it might be good to connect the terms tailbud and post-tailbud stages to the more general conventions of segmentation and pharyngula stages (https://zfin.org/zf_info/zfbook/stages/) since I am not aware of the general use of the term “tailbud stages” in zebrafish.

© 2021. Published by The Company of Biologists under the terms of the Creative Commons Attribution License (https://creativecommons.org/licenses/by/4.0/).

Page 5 para 1 In this paper the authors say that notochord cells begin to move posteriorly in post-tailbud stages due to vacuolation of the cells. Since Ellis, 2013 says that vacuolation begins considerably earlier (17 hpf), is the movement really only beginning at this later stage (implying that there has to be enough vacuole expansion to trigger it) or is it that it just becomes more noticeable in the post-tailbud stages? If the former then the current text is fine.

Page 5 para 2 Either in the text or legend it should state the stage the ablation was done.

Page 7 para 1 I am not sure what the authors mean by “almost complete notochord ablation”.

Looking at the beginning of movie 5 it looks to me like there is quite a bit of residual notochord. It would help to be more precise and state the approximate percent of the total notochord that was ablated at the start of the movie.

Page 9 para 1. The current name for flh is noto. More importantly, I don’t think the authors have shown “regeneration” especially as they have not shown after ablation that they have completely eliminated noto expression. Since regeneration has a very specific meaning in developmental biology involving proliferation from some sort of progenitor pool followed by differentiation to a specific type (in this case notochord cells), I think it would be much better to use the word “recovery” rather than regeneration. If they want to claim regeneration, I think much more work is needed to show that.

Reviewer 3

Advance summary and potential significance to field

In this work, the authors demonstrate a role of notochord morphogenesis in body axis elongation. Specifically, mechanical action derived from the notochord plays an important role in the morphogenesis of coupled paraxial tissues (referred to by the authors as the somitic compartment).

The authors report that in post-tailbud growth stages, segmented tissue elongates due to an A-P stretch caused by a pushing force from progressive notochord cell expansion. The presence of this pushing force is assayed by multi-photon laser ablation to remove notochord cells in a spatially restricted manner. Cells located posterior to ablation site of anterior notochord cells (expanded) displace anteriorly, in contrast to non-ablated embryos Â– indicating that these cells experience a posterior-ward deformation.

To generate the A-P stretch on the segmented tissue, the authors suggest that the posterior not-yet-vacuolated cells must resist the push from notochord cell expansion. Here, by ablating posterior notochord cells, they demonstrate that cells anterior to the ablation displace posteriorly, indicating that posterior notochord cells resist the force. They also use drug treatments to inhibit notochord vacuolation.

In both ablation perturbation schemes, segmented tissue is shorter by 0.5-1 segment length, compared to control. Other interesting observations presented are the robustness of posterior axis elongation to anterior ablation of the posterior cells and the ablation of notochord progenitors.

The strength of the manuscript is in the ablation experiments and the subsequent quantification. Interpretation of the results are consistent with data presented here and in general support the model proposed to support the role of notochord morphogenesis in post-tailbud growth of the segmented tissue of the somitic compartment. However, the aim of the study and therefore their model is inadequately contextualised within the larger literature of vertebrate body axis morphogenesis. Further, a clearer description of results and more careful analysis will strengthen the manuscript.

Comments for the author

Below, major and minor concerns are highlighted.

© 2021. Published by The Company of Biologists under the terms of the Creative Commons Attribution License (https://creativecommons.org/licenses/by/4.0/).

Major concerns
1. The manuscript will be well served by having a paragraph(s) in the introduction and perhaps a supporting schematic figure that describes – 1.
Zebrafish body axis elongation, specifically mentioning elongation and tail straightening phases. 2. Description/definition of what authors mean by "axial tissues" – is this term in reference to derivatives of the axial mesoderm along or does it also include the paraxial mesoderm/somitic compartment? 3. Notochord morphogenesis, including markers such as flh that identify progenitors, the formation of the "stack of coins", medio-lateral convergence-extension (if relevant- abstract not followed up in intro), sequential notochord cell expansion by vacuole inflation 4. Developmental timeline of notochord morphogenesis events, linking to sequential segmentation of the somitic compartment.

2. While the experiments do back up the proposed model, the authors do not comment on the magnitude of the effect and the contextualisation is conservative. The results presented here show that post-tailbud segmented tissue elongation is 32 um and loss of length due to ablation is similar, and therefore a small fraction of total axis length – what is the net contribution to/cumulative effect of the notochord morphogenesis driven elongation of segmented tissue on axis elongation and straightening? Coupling of notochord and somitic tissues (Dray 2013, Tlili 2019) is mentioned, but how these results fit with their model remains unexplored. Furthermore the known axis truncation of mutants that have affected notochord vacuole biogenesis are not discussed (Sun et al. 2020 might be relevant here for their somite shorten mutant).

3. Some discussion, and explicit inclusion into the model presented, of the spatio-temporal specificity of the A-P stretch on segments will also clarify the reported morphogenetic mechanism. As per our understanding of the proposed model, the posterior push and resistance within the notochord will only result in an A-P stretch in a region of coupled tissue located anterior to the push.

Furthermore, is it a possibility that the effect is experienced in a spatially restricted manner- does anterior notochord resistance (if any) to the vacuole expansion generate forces that limit the anterior extent of the stretch felt by segments?

4. An expanded concluding paragraph to providing a more complete model of axis elongation by notochord morphogenesis would strengthen the paper and the discoveries of the authors.

5. The strongest parts of the manuscript arise in Figures 3 and 4. These present new analyses that move forward our understanding of the role of the notochord in tissue morphogenesis. In contrast, Figures 1 and 2 are not particularly novel. Figure 1 is essentially, as highlighted by the authors, a more careful quantification of previous work. Observations of relative tissue movements (e.g. Figure 2E-F) have already been reported in Tlili et al. PNAS 2019. Further, Fig 2D, follows sequential maturation – is this a discovery of this paper or should a study be referenced here? (Dale 2011? Yamamoto 2010?).
Either way, these results need clearer contextualisation.
The most interesting part of Figure 2 is the cell tracking. Yet, very few cells are shown and this does not provide sufficient confidence in the results. It may improve the paper to shorten the first few figures – focusing only on the essential points – and then developing the later results in more detail (as discussed below).

6. Related to the above point, the results in Fig 3G could be developed more clearly. What is the rate of length gain (plot the slopes)? Similar graphs are also required for bafilomycin treatment. It would be interesting to comment on any difference between the two – spatially restricted ablation versus global manipulation of vacuole biogenesis.
Further related to Fig3. It would help to comment on the supposed magnitude of effect from notochord elongation on segmented tissue elongation. Better quantification is required with clearer annotation of where in the body perturbations have been performed and where measurements have been made.

7. "We hypothesised that notochord cell expansion elongated segmented tissue via an AP oriented stretch. This would require notochord cell expansion to be resisted in the AP direction."

© 2021. Published by The Company of Biologists under the terms of the Creative Commons Attribution License (https://creativecommons.org/licenses/by/4.0/).

This is very unclear, with regard to the directionality and symmetry of these proposed effects. This opening to the Figure 4 needs to be improved to make clear what is being tested and what the underlying hypotheses are.
8. “We find that notochord cell expansion generates a force that deforms segmented tissue during post-tailbud stages of development, contributing to AP axis elongation.” This can be tested with laser ablation. The spatial magnitude of the effect can be quantified (i.e. do all segmented tissues feel this stretch?).

9. In general, the statistical analysis of the results feels underpowered. For example, in the tracks it is often a single cell from a single embryo. This is ripe for bias in human choice of cell. Of course, with Covid disruptions, it is hard to collect large quantities of data. But, it would be good to show a larger number of cells from each embryo to show clearly that the observed behaviour is consistent. Further, ablation experiments are known to be quite heterotypic.
Therefore, n=6 or 7 in Figure 3B is likely significantly underpowered to make a decision regarding statistical significance. The authors should perform a power analysis on how many experiments should be needed to make a robust estimate of the significance. I suspect further experiments will be required.

10. The figure quality is generally quite poor in terms of resolution and clarity.
This may be due to a conversion issue, but the authors should look to improve resolution and also labelling of figures (see comments below).

Minor comments
1. Please add lines numbers – it makes it much easier to give specific comments.
2. Introduction: What do multiple axial tissues mean? This needs clearer definition.
3. Fig S1 – Exactly how are trunk and tail defined? Provide schematic to define.
4. What is the definition of adjacent axial tissues? Again, schematic may help.
5. Fig 2E,F – what’s the rationale for 5 hours? What if left longer? What is the maximal relative displacement?
6. Fig 2G,H – where are the panels from with reference to the body and ablation site? Where are the measurements taken? Some explanation, albeit insufficient comes in the paragraph supporting Fig3. Can this be specified in actual segment numbers and anatomical landmarks?
7. Fig 3E – where are the measured segments, relative to ablation site?
8. Fig 3H – what is the segment phenotype?
9. Fig S3D – what is the green label?
10. Fig 4 – Specify more clearly the locations relative to ablation. In the accompanying text – “anterior; locations of anterior ablations” - can this be specified in actual somite numbers and anatomical landmarks?
11. Fig 4C – polar plot – which population is which? Annotate
12. Fig 4E – errors to annotate movement directions will be helpful.
13. Fig 4F – where are segments shown relative to the ablation site? Location in body same as 4E?

First revision

<u>Author response to reviewers' comments</u>

Response to reviewers

We would like to thank each of the reviewers for their insightful and constructive feedback. We have made changes to the text and figures to address these points, improving the quality of the manuscript. Please find our responses to each of the points raised below.

Reviewer 1 Advance summary and potential significance to field

In the manuscript by McLaren and Steventon the authors investigated the role of the notochord in axis elongation in zebrafish. Using quantitative microscopy methods and cells ablation as main

© 2021. Published by The Company of Biologists under the terms of the Creative Commons Attribution License (https://creativecommons.org/licenses/by/4.0/).

experimental tool they showed that vacuolated cell expansion at the anterior of the notochord, resisted by the undifferentiated posterior end contributes to axis elongation. While some of the main conclusions were previously reached qualitatively by other studies, their quantitative analyses add precision and suggest a mode and timing of tissue-tissue coupling during axis elongation that is novel. Particularly interesting are their conclusions about the role of the notochord in the expansion of segmented tissue as it shows an aspect of notochord-dependent axis elongation that has not previously been examined in any detail and was somewhat dismissed by other studies focused on posterior cell addition.

Reviewer 1 Comments for the author

Overall, this is an interesting and straightforward study that reaches some clear conclusions and opens some questions for future studies. However, there are technical limitations due to the lack of specific markers, a need to perform additional controls, address experimental conditions and perhaps reconsider some discussion points. Some technical details and figure labelling also need attention.

Major points:

1-The authors always refer to notochord cells throughout the manuscript and do not make distinction between vacuolated and sheath cells. Due to the absence of specific markers for each population they cannot always tell if they are following or targeting vacuolated cells, sheath cells or the undifferentiated tissue. For example, the relative displacement of notochord and somites, Fig.2EF, and the cells followed in 2G likely are vacuolated cells. In contrast, in the undifferentiated tissue it is not possible to distinguish both populations and the whole rod may be displaced by the expansion of vacuoles more anteriorly. Whenever appropriate vacuolated cells need to be mentioned specifically as the relative movement of vacuolated cells due to vacuole expansion is also relative to the sheath epithelium in the anterior portion of the notochord. If the authors suggest the whole tissue, including the sheath epithelium, is being displaced they would need to use specific markers and more detailed observations. For specific markers see Yamamoto et al. (2010, PMID 20573700); Garcia et al. (2017, PMID 28648824); Dale et al. (2011, PMID 21723274).

In order to follow tissue displacements across the whole-body axis, our imaging is not at sufficient resolution to determine whether or not the vacuolated cells move together with the sheath cells or not. Therefore, the reviewer is correct in saying that we can only conclude about the relative shift of vacuolated cells tracked in the anterior, and undifferentiated cells tracked in the posterior. Cells in Fig.2F were tracked within the centre of the notochord and underwent expansion over the duration of the track. Cells tracked in the posterior were located in the undifferentiated region of the notochord, and therefore tracks in this region may include cells fated to become either vacuolated or sheath cells. We only show that these cells do not move as much relative to adjacent somites as cells in the more anterior expanded regions of the notochord, and that they provide resistance to anterior cell expansion.

Specification of whether vacuolated notochord cells, or cells in the undifferentiated region were followed has been added throughout the text. Ablations were carried out in embryos at 16-18 somite stages and may have included undifferentiated cells fated to become sheath cells, in addition to the vacuolated cell type. We have clarified this point in the methods section – lines 592 to 596 (as the restricted word limit does not accommodate for this in the main text).

2-The labeling experiment in Fig.2EF is both relatively simple and compelling. Perhaps if it is repeated at different AP levels and developmental windows we may learn more about the degree of tissue-tissue coupling, which is discussed later in the manuscript.

We thank the reviewer for this suggestion and have now included this additional data for tissue shifts in post-tailbud stage embryos in a revised Fig. 2 of the manuscript. The shift between notochord-somite and notochord-neural tube labels decreases with decreasing distance from the posterior end of post-tailbud stage embryos (Figs. 2A- C). This is in agreement with our tracking data – showing that notochord cell displacement relative to an adjacent somite boundary is lower in the posterior and higher in the anterior (Figs.2G and H).

© 2021. Published by The Company of Biologists under the terms of the Creative Commons Attribution License (https://creativecommons.org/licenses/by/4.0/).

A shared interface of extracellular matrix between the somitic compartment and notochord is thought to physically couple these two tissues (Dray et al. 2013; Guillon et al. 2020). We have investigated notochord cell adhesion to this interface in the anterior and posterior of post-tailbud stage embryos and added our findings to the manuscript and our model – Figs.4 and S6, lines 233-236.

3-In the experiments presented in figure 3 and S3 they authors show a role for vacuolated cell expansion in segmented tissue that is highly interesting. However, they also present data in figure S3 that suggest a role in axis straightening that needs to be discussed critically as mutants with vacuole fragmentation or conditions in which vacuolated cell number are reduced lead to significant shortening of the axis without clear defects in straightening. Therefore, it is possible that the relatively mild straightening differences they observed are due to tissue damage rather than a direct result of a loss of notochord stiffness.

We agree that this is a possibility, as the region used for curvature measurements includes the region of the axis in which ablation was performed, and therefore the curvature may be influenced by a transient response to the ablation itself, rather than a consequence of notochord displacement. For this reason, and because this data is not a main part of our findings, we will remove this analysis from the manuscript.

4-Could the authors show controls for the extent of damage produced by the ablations? Controls for potential damage to other tissues are also needed.

To investigate the extent of any possible off-target tissue damage caused by notochord ablations we investigated Shh expression in the floorplate lying dorsal to an ablated notochord region (in ablated embryos) and intact notochord region (in control embryos) using HCR. Notochord cells were ablated and embryos fixed approximately 1.5 hours post-ablation. Shh expression appears to be normal in the floorplate overlying the ablated area, indicating that surrounding tissues are not majorly damaged by notochord ablations (Fig. 2D).

5-Following ablation, cells (presumably vacuolated) appear to be displaced anteriorly (Fig.2G). Are cells filling the space of the ablated cells? How is this response interpreted?

Yes, expanding cells move into the space created by the ablation (Movie 4)

6-In figure a higher n is would be best for nuclei angle measurements.

5 more ablated embryos and 5 more control embryos have been added to this analysis (with 5 nuclear angle measurements per embryo) (Fig.4B and S6A).

Minor points:
7-One aspect that may need to be considered further is the interpretation of the role of the unexpanded notochord in resisting the force from anterior expansion. Even at early larval stages expansion continues at the posterior end and this process has an impact in axis elongation that is apparent in mutants, e.g. cavin1b (see Garcia et al. 2017, PMID 28648824; and Lim et al. 2017, PMID28648821), in which vacuolated cell disruption occurs after hatching. The extrusion of vacuolated cells seen in Romero et al (2018) and also Norman et al. (2018, PMID30249771) reflects internal pressure held by the sheath that was severed rather than a loss of resistance from the unexpanded notochord. This also brings up the need to consider the development of hydrostatic pressure throughout the notochord as vacuoles expand within the sheath that couples the whole structure.

We agree with the reviewer that at much later stages, notochord expansion continues to propagate into the tail and will eventually remove any posterior resistance generated from unvacuolated cells. As cell expansion progresses posteriorly along the notochord and the progenitors are depleted over time, it may be that eventually the entire length of the notochord will become vacuolated. We have looked up until ~10 hours post-segmentation stages and observe that, whilst much of the posterior notochord has become vacuolated, unexpanded cells still make up the most posterior region of the notochord (Rebuttal Fig.1), and therefore may still be

© 2021. Published by The Company of Biologists under the terms of the Creative Commons Attribution License (https://creativecommons.org/licenses/by/4.0/).

providing resistance to anterior cell expansion at these stages. However, the reviewer is correct that it may be the sheath cells, and not the vacuolating cells themselves that are providing posterior resistance at later stages. During the time period that we are considering, posterior notochord cells are still in an unexpanded state and will only later differentiate into the sheath and/or vacuolated notochord. Therefore, we do not distinguish between these possibilities in the context of this work.

8-While the effect of vacuolated cell ablation is likely not confounded by regenerative responses during the short time frame of their experimental setup, it is worth considering whether expansion of neighboring intact vacuolated cells and changes in cell packing (see Norman et al., PMID 30249771 and Garcia et al. 2017, PMID 28648824) may dampen the effect of cell ablation.

We agree with the reviewer that the effect of cell ablation is likely to be dampened. The reversed displacement of expanding vacuolated cells effectively acts to 'close the gap' in ablated embryos. Thus, the impact of ablations is transient (Movie 4).

(lines 137-138).

9-Markers in figures S3 (ntl:kaede?) and S4 (HCR for flh?) and not described.

Description labels have been added to figures 3I and S5A.

10-Some details about the photolabeling experiments would be useful.

The photolabeling experiment is now clearly mentioned in the text (line 120) and described in more detail in the methods (lines 557-560) and in figure S3.

Reviewer 2 Advance summary and potential significance to field
The notochord has long been known to have an important role in the morphogenesis of the AP axis, shown nicely in zebrafish with a variety of mutants that affect the formation of the notochord. The authors here study the role of the notochord in axis elongation during the somitogenesis stages, which has not been well characterized, using targeted laser ablation. They nicely show that a combination of the anterior notochord expanding through vacuolation and providing a posterior directed force together with addition of cells from the posterior progenitors, drives the morphogenetic events.

The work is beautifully done and well presented, and provides a much more careful understanding of the role of the notochord in vertebrate morphogenesis. The comments are just some suggestions to improve the text.

Reviewer 2 Comments for the author
Page 2, para 2 It seems a bit surprising to me that the noto mutant is not mentioned since it clearly shows in zebrafish how the absence of a notochord affects early embryo morphogenesis. It also shows that the notochord is having effects even by 24 hpf (what the authors call the end of the tailbud stages as shown for example in Talbot, 1995, Fig 1), whereas this paper makes it seem like the notochord only begins to have effects on morphogenesis after this stage. I think it would help to briefly integrate the pre-24 hpf effects in the text.

We agree with the reviewer that there are defects in somite shape and length in the noto mutant shown in the Talbot et al. paper. In this case the lack of notochord causes somites to fuse medially under the neural tube. Therefore, it is it is likely the fusion of somites due to the lack of a partitioning tissue, rather than a change in notochord morphogenesis, leads to axis elongation defects at these earlier stages.

In addition, the posterior-body elongation defects observed at 24hpf are relatively mild considering that the entire notochord is missing in agreement with our finding that segmentation-derived elongation is robust to defects in notochord morphogenesis.

We have now cited this mutant and paper in the main text (line 50).

© 2021. Published by The Company of Biologists under the terms of the Creative Commons Attribution License (https://creativecommons.org/licenses/by/4.0/).

Page 3, para 2 With regards to the statement about "boundaries of 5 formed somites" is that 5 somites picked randomly or the same 5 somites in all embryos (ie somites 1-5)? It would help to be a bit more specific.

We thank the reviewer for this suggestion. Schematics have now been added to show the ablated region and region of somites used for elongation measurements for all experiments (Figs. 1A, 3A and E and 4C).

Page 3, para 3 It might be a bit better to say "starting at" rather than "during". I also think it might be good to connect the terms tailbud and post-tailbud stages to the more general conventions of segmentation and pharyngula stages (https://zfin.org/zf_info/zfbook/stages/) since I am not aware of the general use of the term "tailbud stages" in zebrafish.

We make use of the term 'tailbud stages' to emphasise the continued existence of undifferentiated progenitors in the posterior that will provide an additional source of cells for axial elongation. A link between tailbud and post-tailbud stages and the segmentation and the pharyngula period has been added to the methods section – lines 527-529.

Page 5 para 1 In this paper the authors say that notochord cells begin to move posteriorly in post-tailbud stages due to vacuolation of the cells. Since Ellis, 2013 says that vacuolation begins considerably earlier (17 hpf), is the movement really only beginning at this later stage (implying that there has to be enough vacuole expansion to trigger it) or is it that it just becomes more noticeable in the post-tailbud stages? If the former, then the current text is fine.

Yes, cells in regions where notochord cells are yet to expand in post-tailbud stage embryos move relatively little to surrounding tissues (Fig.2A-C, and H). We take this to mean that it requires a certain degree of build-up in vacuolated cells to enable a posterior displacement at the tissue level.

Page 5 para 2 Either in the text or legend it should state the stage the ablation was done.

This information has been added to the figure legend (Fig.S2).

Page 7 para 1 I am not sure what the authors mean by "almost complete notochord ablation". Looking at the beginning of movie 5 it looks to me like there is quite a bit of residual notochord. It would help to be more precise and state the approximate percent of the total notochord that was ablated at the start of the movie.

This has been clarified as 'an ablation that extended into the posterior notochord' (line 154).

Page 9 para 1. The current name for flh is noto. More importantly, I don't think the authors have shown "regeneration" especially as they have not shown after ablation that they have completely eliminated noto expression. Since regeneration has a very specific meaning in developmental biology involving proliferation from some sort of progenitor pool followed by differentiation to a specific type (in this case notochord cells), I think it would be much better to use the word "recovery" rather than regeneration. If they want to claim regeneration, I think much more work is needed to show that.

We agree with the reviewer that more evidence is required to demonstrate a complete regeneration, We have altered the description to 'recovered' as suggested. In addition, we have changed flh to noto throughout the text and figures.

Reviewer 3 Advance summary and potential significance to field
In this work, the authors demonstrate a role of notochord morphogenesis in body axis elongation. Specifically, mechanical action derived from the notochord plays an important role in the morphogenesis of coupled paraxial tissues (referred to by the authors as the somitic compartment).

The authors report that in post-tailbud growth stages, segmented tissue elongates due to an A-P stretch caused by a pushing force from progressive notochord cell expansion. The presence of this pushing force is assayed by multi-photon laser ablation to remove notochord cells in a spatially

© 2021. Published by The Company of Biologists under the terms of the Creative Commons Attribution License (https://creativecommons.org/licenses/by/4.0/).

restricted manner. Cells located posterior to ablation site of anterior notochord cells (expanded) displace anteriorly, in contrast to non-ablated embryos – indicating that these cells experience a posterior-ward deformation.
To generate the A-P stretch on the segmented tissue, the authors suggest that the posterior not-yet-vacuolated cells must resist the push from notochord cell expansion. Here, by ablating posterior notochord cells, they demonstrate that cells anterior to the ablation displace posteriorly, indicating that posterior notochord cells resist the force. They also use drug treatments to inhibit notochord vacuolation.

In both ablation perturbation schemes, segmented tissue is shorter by 0.5-1 segment length, compared to control. Other interesting observations presented are the robustness of posterior axis elongation to anterior ablation of the posterior cells and the ablation of notochord progenitors.

The strength of the manuscript is in the ablation experiments and the subsequent quantification. Interpretation of the results are consistent with data presented here and in general support the model proposed to support the role of notochord morphogenesis in post-tailbud growth of the segmented tissue of the somitic compartment. However, the aim of the study and therefore their model is inadequately contextualised within the larger literature of vertebrate body axis morphogenesis. Further, a clearer description of results and more careful analysis will strengthen the manuscript.

Reviewer 3 Comments for the author
Below, major and minor concerns are highlighted.

Major concerns

1.The manuscript will be well served by having a paragraph(s) in the introduction and perhaps a supporting schematic figure that describes – 1. Zebrafish body axis elongation, specifically mentioning elongation and tail straightening phases. 2. Description/definition of what authors mean by “axial tissues” – is this term in reference to derivatives of the axial mesoderm along or does it also include the paraxial mesoderm/somitic compartment? 3. Notochord morphogenesis, including markers such as flh that identify progenitors, the formation of the “stack of coins”, medio-lateral convergence-extension (if relevant- abstract not followed up in intro), sequential notochord cell expansion by vacuole inflation 4. Developmental timeline of notochord morphogenesis events, linking to sequential segmentation of the somitic compartment.

We thank the reviewer for this suggestion. Introductory schematics and legends containing accompanying information have been added to figure 1. Segmented tissue elongation (orange) and segmentation-derived elongation (blue) is clarified in 1A. 1B clarifies the axial tissues (somitic compartment and neural tube) we refer to in the main text and their organisation relative to the notochord. 1C depicts events during notochord morphogenesis.

2.While the experiments do back up the proposed model, the authors do not comment on the magnitude of the effect and the contextualisation is conservative. The results presented here show that post-tailbud segmented tissue elongation is 32 um and loss of length due to ablation is similar, and therefore a small fraction of total axis length – what is the net contribution to/cumulative effect of the notochord morphogenesis driven elongation of segmented tissue on axis elongation and straightening?

To estimate the magnitude of the impact of notochord ablation on overall somitic compartment elongation we can extrapolate our findings to a timepoint when the entire notochord has undergone vacuolation and estimate the prolonged impact of the decrease we observe on the whole somitic compartment. The entire notochord appears to be fully vacuolated by 3dpf (Kimmel et al. 1995), by this point we would expect notochord morphogenesis to impact the whole length of the somitic compartment.

Extrapolating to a scenario in which the impact of notochord ablation on somite elongation was maintained over a 2-day time period after the onset of increased segmented tissue elongation (starting ~1dpf) we can estimate the magnitude of the impact of notochord ablation on somitic compartment elongation. However, this assumes that all somites are impacted equally and

© 2021. Published by The Company of Biologists under the terms of the Creative Commons Attribution License (https://creativecommons.org/licenses/by/4.0/).

elongate at the same rate as our measured region, and our current knowledge of the changing mechanical properties of the somitic compartment is not yet sufficient to comment on this in the context of the paper with a sufficient degree of certainty. Nevertheless, using equations for the rate of somite region elongation in control and ablated embryos obtained from linear regression analysis gives us an expected decrease in the overall length of the somitic compartment of 28% in ablated embryos compared to controls, though this is likely an overestimate given the observed ability of axial tissues to regulate upon tissue loss. This is in broad agreement with experimental findings showing that embryos with mutations affecting notochord vacuolation have body lengths that are 17.5% shorter at 3-days post fertilisation {Sun:2020jq}. This leads to severe scoliosis at adult stages of development. We have cited this article in our revised conclusions section, as we feel this mutant phenotype best reflects the phenotypic impact of a cumulative effect of the lack of notochord vacuolation. Our ablations allow us to target notochord cell expansion in a spatially and temporally specific way and measure the subsequent mechanical impact on somite elongation.

Coupling of notochord and somitic tissues (Dray 2013, Tlili 2019) is mentioned, but how these results fit with their model remains unexplored. Furthermore the known axis truncation of mutants that have affected notochord vacuole biogenesis are not discussed (Sun et al. 2020 might be relevant here for their somite shorten mutant).

We have added points to the discussion outlining how tissue coupling fits within our model (lines 233 – 244). We have also indicated the zone of stronger tissue coupling in the posterior in Fig. 4J.

The Sun et al. mutant is now cited in the discussion (line 228).

3.Some discussion, and explicit inclusion into the model presented, of the spatio-temporal specificity of the A-P stretch on segments will also clarify the reported morphogenetic mechanism. As per our understanding of the proposed model, the posterior push and resistance within the notochord will only result in an A-P stretch in a region of coupled tissue located anterior to the push. Furthermore, is it a possibility that the effect is experienced in a spatially restricted manner- does anterior notochord resistance (if any) to the vacuole expansion generate forces that limit the anterior extent of the stretch felt by segments?

Spatial specificity of the AP stretch is now discussed in lines 240 – 246. Since vacuolation progresses posteriorly along the notochord, we expect that the posterior region resisting anterior cell expansion will shrink over time. This corresponds to the region of stronger coupling between notochord cells and the somitic compartment. Thus more posterior regions of the somitic compartment will be exposed to a stretch as development progresses.
Anterior resistance to notochord cell expansion is likely provided by a tissue boundary at the anterior extent of the notochord, which also aligns with the anterior extent of the somitic compartment. Due to word limit constraints, we have not included this more speculative aspect in the discussion.

4.An expanded concluding paragraph to providing a more complete model of axis elongation by notochord morphogenesis would strengthen the paper and the discoveries of the authors.

A more complete description of the model and discussion of how this relates to axis elongation has been incorporated into the second to las paragraph (lines 224-246).

5.The strongest parts of the manuscript arise in Figures 3 and 4. These present new analyses that move forward our understanding of the role of the notochord in tissue morphogenesis. In contrast, Figures 1 and 2 are not particularly novel. Figure 1 is essentially, as highlighted by the authors, a more careful quantification of previous work. Observations of relative tissue movements (e.g. Figure 2E-F) have already been reported in Tlili et al. PNAS 2019. Further, Fig 2D, follows sequential maturation – is this a discovery of this paper or should a study be referenced here? (Dale 2011? Yamamoto 2010?). Either way, these results need clearer contextualisation.

© 2021. Published by The Company of Biologists under the terms of the Creative Commons Attribution License (https://creativecommons.org/licenses/by/4.0/).

The most interesting part of Figure 2 is the cell tracking. Yet, very few cells are shown and this does not provide sufficient confidence in the results. It may improve the paper to shorten the first few figures – focusing only on the essential points – and then developing the later results in more detail (as discussed below).

Figures 1 and 2 have been reworked to focus on more novel findings of this study, and results confirming findings in previous studies have been moved to supplementary figure 2.
Findings shown by Tlili et al. are cited in the discussion (line 230). In Fig. 2 we show that notochord cell expansion (due to vacuolation) in post-tailbud stages leads to the posterior displacement of expanded notochord cells relative to adjacent tissues – showing a different cause of relative tissue movements to those in Tlili et al.
We have referenced studies showing that vacuolated cells expand over time and have added citations for papers from Dale and Yamamoto (line 118).

The number of cells tracked in embryos with anterior notochord ablations and controls has been increased to include 3 tracks per region, per embryo. In addition, tracks from more embryos have been added to the plots. See Figs. 2G and H.

6.Related to the above point, the results in Fig 3G could be developed more clearly. What is the rate of length gain (plot the slopes)? Similar graphs are also required for bafilomycin treatment. It would be interesting to comment on any difference between the two – spatially restricted ablation versus global manipulation of vacuole biogenesis. Further related to Fig3. It would help to comment on the supposed magnitude of effect from notochord elongation on segmented tissue elongation. Better quantification is required with clearer annotation of where in the body perturbations have been performed and where measurements have been made.

Linear regression analysis has been performed and the corresponding equations with slope coefficients have been added to the plots (Figs. 3D, H and S4C). The impact of global inhibition of vacuolation with Bafilomycin versus spatially restricted notochord cell ablation has been added to the text (lines 169-170).

Schematics have been added throughout the manuscript showing the regions where ablations were performed and where measurements have been made (Figs.2E, 3A and E, and 4C).

7.“We hypothesised that notochord cell expansion elongated segmented tissue via an AP oriented stretch. This would require notochord cell expansion to be resisted in the AP direction.” This is very unclear, with regard to the directionality and symmetry of these proposed effects. This opening to the Figure 4 needs to be improved to make clear what is being tested and what the underlying hypotheses are.

This has been clarified in the text (lines 176-18). For a stress to be generated in the AP direction, expanding notochord cells need something to push against. We test whether the posterior displacement of expanding vacuolated notochord cells is resisted by unexpanded cells in the posterior notochord.

8.“We find that notochord cell expansion generates a force that deforms segmented tissue during post-tailbud stages of development, contributing to AP axis elongation.” This can be tested with laser ablation. The spatial magnitude of the effect can be quantified (i.e. do all segmented tissues feel this stretch?).

In this work, our conclusions relating to the impact of notochord expansion on the AP elongation of segmented tissue is focussed on a phenotypic analysis of the phenomenon. We observe a decrease in somitic tissue elongation in response to a disruption of notochord cell expansion. Whether this is a direct force transmission that leads to the stretching of myofibers to elongate them, or alternatively an indirect mechanism via the regulation of myogenesis has not been determined. The experiments suggested by the reviewer: laser ablation within the somitic tissue to measure direct force transmission would help to resolve this point. However, many additional experiments would also be required to rule out the alternative hypothesis of an indirect regulation via

© 2021. Published by The Company of Biologists under the terms of the Creative Commons Attribution License (https://creativecommons.org/licenses/by/4.0/).

differentiation. This is an important question for future studies and has now been highlighted in the closing lines of the discussion (lines 250-254).

9.In general, the statistical analysis of the results feels underpowered. For example, in the tracks it is often a single cell from a single embryo. This is ripe for bias in human choice of cell. Of course, with Covid disruptions, it is hard to collect large quantities of data. But, it would be good to show a larger number of cells from each embryo to show clearly that the observed behaviour is consistent.

More tracks per embryo and more embryos have been added to the analysis as detailed in revision point 5. Notochord cell movement is consistent within tracked regions. This can also be observed in supplementary movies 3 and 7.

Further, ablation experiments are known to be quite heterotypic.
Therefore, n=6 or 7 in Figure 3B is likely significantly underpowered to make a decision regarding statistical significance. The authors should perform a power analysis on how many experiments should be needed to make a robust estimate of the significance. I suspect further experiments will be required.

A power analysis has been conducted to detect a difference between the means of each group of 3% at a standard power score of 0.8. Using the standard deviation of each initial sample, an effect size of 1.104 was calculated. Power analysis indicated that a sample size of 13 control and 11 ablated embryos was required. This has now been achieved (14 control and 11 ablated embryos measured in total), see figs. 3C and D.

A non-significant p value was obtained (p=0.37) with this analysis. In addition, a separate analysis on embryos with notochord progenitor ablations (fig. S5), also indicates that segmentation-derived tail elongation can continue in the absence of a continuous notochord. Together these results support our conclusion that segmentation-derived tail elongation is robust to perturbation of notochord cell expansion.

10.The figure quality is generally quite poor in terms of resolution and clarity.
This may be due to a conversion issue, but the authors should look to improve resolution and also labelling of figures (see comments below).

Plots have been saved in .png format to increase figure quality.

Minor comments

1.Please add lines numbers – it makes it much easier to give specific comments.

Line numbers have been added.

2.Introduction: What do multiple axial tissues mean? This needs clearer definition.

The neural tube and somitic compartment are the axial tissues mentioned. This has been clarified with a schematic (fig.1B)

3.Fig S1 – Exactly how are trunk and tail defined? Provide schematic to define.

Trunk and tail analyses are no longer included in the manuscript due to previous revision points.

4.What is the definition of adjacent axial tissues? Again, schematic may help.

These are now referred to in the text – line 122.

5.Fig 2E,F – what's the rationale for 5 hours? What if left longer? What is the maximal relative displacement?

© 2021. Published by The Company of Biologists under the terms of the Creative Commons Attribution License (https://creativecommons.org/licenses/by/4.0/).

A 5-hour shift was measured so that these findings can be related to our 5-hour somite elongation measurements in ablated embryos.

6.Fig 2G,H – where are the panels from with reference to the body and ablation site? Where are the measurements taken? Some explanation, albeit insufficient comes in the paragraph supporting Fig3. Can this be specified in actual segment numbers and anatomical landmarks?

This has been clarified with schematics showing the location of ablations images showing the location of tracked cells relative to the ablation site (Figs. 2E and F).

7.Fig 3E – where are the measured segments, relative to ablation site?

This has been clarified with schematics showing the location of ablations and measured regions (Fig. 3E).

8.Fig 3H – what is the segment phenotype?

The phenotype of somites in bafilomycin and dmso treated embryos is shown in fig. 3I.

9.Fig S3D – what is the green label?

Label description added to figure 3I (Ntl:Kaede).

10.Fig 4 – Specify more clearly the locations relative to ablation. In the accompanying text – “anterior; locations of anterior ablations” - can this be specified in actual somite numbers and anatomical landmarks?

This has been clarified with schematics showing the location of ablations and measured regions (Fig. 4C).

11.Fig 4C – polar plot – which population is which? Annotate

Annotations have been added (Fig.4B).

12.Fig 4E – errors to annotate movement directions will be helpful.

Arrows have been added (Fig.4D).

13.Fig 4F – where are segments shown relative to the ablation site? Location in body same as 4E?

This has been clarified with schematics showing the location of ablations and measured regions (Fig.4C).

<u>Second decision letter</u>

MS ID#: DEVELOP/2021/199459

MS TITLE: Anterior expansion and posterior addition to the notochord mechanically coordinate embryo axis elongation

AUTHORS: Susannah B P McLaren and Benjamin J Steventon
ARTICLE TYPE: Research Report

I am happy to tell you that your manuscript has been accepted for publication in Development, pending our standard ethics checks. The referee reports on this version are appended below and

© 2021. Published by The Company of Biologists under the terms of the Creative Commons Attribution License (https://creativecommons.org/licenses/by/4.0/).

you will see the referees have a couple of suggestions for minor changes to the text that you might want to incorporate into your final version.

Reviewer 1

Advance summary and potential significance to field

In their manuscript, McLaren and Steventon used quantitative approaches to study the role of the notochord in axis elongation during zebrafish embryogenesis. Their work adds precision to previous studies from other labs and identified specific contributions and interactions between notochord and paraxial mesoderm tissues that collectively play a role in axis elongation. Overall, this is an interesting paper that contributes to our understanding of axis morphogenesis in vertebrates.

Comments for the author

The authors have made substantial revisions that included both experimental and editorial changes that collectively improved significantly the manuscript. They have also answered all points previously raised. Even though there is some uncertainty in the ablation method stemming from the lack of specific makers for notochord cell populations, the authors acknowledged the limitations in the manuscript appropriately. The manuscript is overall greatly improved and should be of interest to the broad readership of Development.

One small editorial point to fix is that Bagwell et al (2020) should be added to the reference of Sun et al in line 228 as both papers reported the same gene and covered the same developmental windows. Moreover Bagwell et al contains more quantitative data relevant for this manuscript.

Reviewer 2

Advance summary and potential significance to field

The notochord has long been known to have an important role in the morphogenesis of the AP axis, shown nicely in zebrafish with a variety of mutants that affect the formation of the notochord. The authors here study the role of the notochord in axis elongation during the somitogenesis stages, which has not been well characterized, using targeted laser ablation. They nicely show that a combination of the anterior notochord expanding through vacuolation and providing a posterior-directed force together with addition of cells from the posterior progenitors, drives the morphogenetic events.

Comments for the author

The authors have addressed my concerns for the most part. Personally, I think it would be better to have the definition of tailbud stages and post-tailbud stages in the main text rather than buried in the Methods (where they have now added it) since these are not commonly used terms that I am aware of and I think for most readers they will not think to look in the Methods to find these definitions, but I leave that decision to the editor.

Reviewer 3

Advance summary and potential significance to field

This paper advances our understanding of the role of the notochord in axis elongation. It combines quantitative measures with suitable biophysical measures t dissect the underlying mechanical processes.

Comments for the author

The authors have done a very good job in dealing with the concerns raised. The manuscript is substantially improved and the new data is more convincing.

© 2021. Published by The Company of Biologists under the terms of the Creative Commons Attribution License (https://creativecommons.org/licenses/by/4.0/).
